# Identifying Dysfunctional Cortex: Dissociable Effects of Stroke and Aging on Resting State Dynamics in MEG and fMRI

**DOI:** 10.3389/fnagi.2016.00040

**Published:** 2016-03-03

**Authors:** Aneta Kielar, Tiffany Deschamps, Ron K. O. Chu, Regina Jokel, Yasha B. Khatamian, Jean J. Chen, Jed A. Meltzer

**Affiliations:** ^1^Rotman Research Institute, Baycrest Health SciencesToronto, ON, Canada; ^2^Department of Psychology, University of TorontoToronto, ON, Canada; ^3^Department of Speech-Language Pathology, University of TorontoToronto, ON, Canada; ^4^Department of Medical Biophysics, University of TorontoToronto, ON, Canada; ^5^Canadian Partnership for Stroke RecoveryOttawa, ON, Canada

**Keywords:** aging, aphasia, stroke, MEG, blood flow, BOLD variability, multiscale entropy

## Abstract

Spontaneous signals in neuroimaging data may provide information on cortical health in disease and aging, but the relative sensitivity of different approaches is unknown. In the present study, we compared different but complementary indicators of neural dynamics in resting-state MEG and BOLD fMRI, and their relationship with blood flow. Participants included patients with post-stroke aphasia, age-matched controls, and young adults. The complexity of brain activity at rest was quantified in MEG using spectral analysis and multiscale entropy (MSE) measures, whereas BOLD variability was quantified as the standard deviation (SD_BOLD_), mean squared successive difference (MSSD), and sample entropy of the BOLD time series. We sought to assess the utility of signal variability and complexity measures as markers of age-related changes in healthy adults and perilesional dysfunction in chronic stroke. The results indicate that reduced BOLD variability is a robust finding in aging, whereas MEG measures are more sensitive to the cortical abnormalities associated with stroke. Furthermore, reduced complexity of MEG signals in perilesional tissue were correlated with hypoperfusion as assessed with arterial spin labeling (ASL), while no such relationship was apparent with BOLD variability. These findings suggest that MEG signal complexity offers a sensitive index of neural dysfunction in perilesional tissue in chronic stroke, and that these effects are clearly distinguishable from those associated with healthy aging.

## Introduction

Cerebrovascular stroke is a common cause of cognitive, language, and motor impairments. Although lesion location is closely related to functional impairments, there is high individual variability among patients with similar lesions in terms of the degree of impairment and recovery outcomes. One reason for this variability may be that ischemic damage extends beyond the primary infarct zone into neuroanatomically intact adjacent cortex. Noninvasive methods are greatly needed to reveal the entire extent of neural dysfunction, especially as dysfunction outside the primary infarct zone may be reversible.

Because stroke-induced infarcts are permanent, recovery is assumed to occur as a result of cortical reorganization and neuroplastic changes that take place in structurally intact brain tissue (Thompson, 2000; Angrilli and Spironelli, 2005; Saur et al., 2006; Thompson and den Ouden, 2008). It is generally thought that recovery of function in perilesional areas offers the best prognosis for clinical improvement after stroke (Heiss et al., 1999; Léger et al., 2002; Heiss and Thiel, 2006). This conclusion is supported by recent studies using transcranial magnetic stimulation (TMS), which have shown improved language performance after inhibitory stimulation to certain contralesional right hemisphere (RH) regions (Naeser et al., 2005, 2010; Winhuisen et al., 2007; Hamilton et al., 2011), or excitatory stimulation to the preserved left hemisphere (LH) cortex adjacent to the lesion (Baker et al., 2010; Fiori et al., 2011). These findings are consistent with a critical role of perilesional tissue in recovery. Therefore, assessing the functionality of these areas using noninvasive methods is essential to tracking recovery and targeting interventions.

Studies of brain electrical activity in stroke using magnetoencephalography (MEG) and electroencephalography (EEG) have indicated that perilesional tissue can produce an abnormally large amount of high amplitude slow-wave activity, mostly in the range between 1 and 6 Hz (delta and theta waves; Vieth et al., 1996; Butz et al., 2004; Meinzer et al., 2004; Tecchio et al., 2005). The presence of increased slow-wave activity is a marker of subtle neural damage associated with the long-term effects of stroke beyond the primary infarct zone, and may be an indicator of the extent of the “functional lesion.” In stroke, a “functional lesion” can extend well beyond the borders of the primary lesion, affecting an individual's cognitive abilities and capacity for recovery (Tecchio et al., 2006; Meinzer et al., 2007; Laaksonen et al., 2013). Modulation of this slow-wave activity has been linked to the improvement of language functions, and it has been shown to change in response to behavioral interventions (Meinzer et al., 2004, 2007).

Although increased slow-wave activity is one potential indicator of cortical dysfunction in perilesional tissue, other measures also show promise. Multiscale entropy (MSE) is one nonlinear measure of complexity that has been used to analyze EEG/MEG (Costa et al., 2005; Park et al., 2007). MSE comprises estimates of sample entropy (Richman and Moorman, 2000) across different time scales, and is thus suitable for applications in which complexity may differ when considered at various degrees of temporal resolution. Greater complexity is associated with healthy processing, whereas reduced values of MSE have been considered as a marker of dysfunction in a variety of physiological signals (Costa et al., 2005; Norris et al., 2008). In addition, reduced MSE has been reported in aging (Yang et al., 2013), and in various clinical conditions, including traumatic brain injury, tumors, and Alzheimer's disease (de Jongh et al., 2001; Park et al., 2007; Poza et al., 2007; Beharelle et al., 2012).

Spectral slowing is reflected in the MSE measure, in that a signal dominated by a highly periodic low-frequency oscillation will also show low entropy. Recently, we found that perilesional tissue in chronic stroke consistently exhibits both increased delta and theta activity, as well as reduced beta activity and reduced MSE values. Of these measures, MSE was the most sensitive to electrophysiological dysfunction in perilesional tissue (Chu et al., 2015). The finding of decreased entropy in perilesional areas suggests that MSE can be a useful marker of cortical dysfunction in tissue that is structurally intact but not functioning optimally.

While questions remain about the best way to quantify neural dynamics with EEG and MEG, other imaging methods also show promise at revealing dysfunction. Several recent studies have analyzed moment-to-moment variability in the blood oxygen level dependent (BOLD) signal of fMRI, as a measure of neural dynamics in resting-state data (Garrett et al., 2010, 2013a). Strong effects have been seen in aging in particular. Garrett et al. (2010) found, that compared to young adults, older adults exhibited less variability in default mode structures and several other regions. Additionally, greater BOLD signal variability was associated with faster, more accurate and consistent performance on various perceptual and cognitive tasks (Garrett et al., 2011, 2013a). BOLD dynamics have also been quantified with sample entropy, showing reduced complexity in aging (Sokunbi, 2014) and ADHD (Sokunbi et al., 2013). Based on these results and others, it was proposed that increased brain signal variability reflects healthier neural dynamics, yielding more stable behavioral performance and the ability to function at a higher cognitive capacity (Ghosh et al., 2008; McIntosh et al., 2008, 2010; Garrett et al., 2013b). These studies suggest that signal variability may be an important index of cognitive functioning.

Therefore, if BOLD signal variability is an index of neuronal robustness, it may serve as an important marker of individual differences in clinical populations. Indeed, previous research established links between brain signal variability and healthy brain function, development, and various clinical conditions, including schizophrenia, traumatic brain injury, epilepsy, and congenital blindness (see Garrett et al., 2013b for review). However, it is unknown whether BOLD variability is altered in perilesional tissue in stroke patients, and how its sensitivity compares with the reduced complexity seen in EEG/MEG.

Another key question is the relationship of resting-state measures to cerebral perfusion. Although the causes of perilesional slow wave activity are not well understood, several mechanisms have been discussed in the literature, including chronic hypoperfusion (Jordan, 2004; Machado et al., 2004; Finnigan et al., 2006), white matter disconnection or deafferentation of cortex (Huang et al., 2009), and ischemic damage caused by the stroke that compromises the functional integrity of the cortex, but does not lead to gross tissue destruction (Claassen et al., 2004; Friedman and Claassen, 2010; Sheorajpanday et al., 2010). These different causes are not independent, as chronic hypoperfusion can lead to neuronal damage and white matter disconnection. Furthermore, the direction of causality can also be reversed; neuronal damage can lead to reduced metabolic demand, reducing perfusion despite an adequate arterial blood supply (Sasaki et al., 1997; Oku et al., 2010). Therefore, sensitive measures of perilesional dysfunction are likely to be correlated with blood flow.

Research from acute and subacute stroke indicates that cerebral blood flow (CBF) is not only disrupted in the neural regions that are directly infarcted, but also in the adjacent cortex (Chalela et al., 2000; Detre, 2001). Decreased CBF in neuroanatomically intact regions has been found to correlate with initial language symptoms in aphasia, as well as short- and long-term recovery success (Mimura et al., 1998; Hillis et al., 2001b,c, 2004a, 2008; Fridriksson et al., 2002; Love et al., 2002). Medical treatment such as pharmacologic or surgical reperfusion of the affected brain regions has been shown to restore language functions in acute and subacute stroke (Hillis et al., 2001a,b, 2004b; Hillis and Heidler, 2002). Although these studies have established that CBF is disrupted in the acute stages after the event, contributing to the cognitive deficits, relatively little research has investigated whether blood flow continues to be abnormal in the chronic stages. Aberrant CBF may endure in the regions that appear anatomically intact (as measured with T1 or T2 weighted clinical MRI), contributing to impaired function and poor recovery outcome (Love et al., 2002). Brumm et al. (2010) used ASL-FLAIR to investigate CBF in three chronic stroke survivors, 2–11 years after stroke. They found regional hypoperfusion in the perilesional areas as well as in the anatomically intact regions, and decreased blood flow was associated with language symptoms. Similarly, Mimura et al. (1998) reported that increased perfusion was related to greater recovery success, even up to 7 years after the stroke.

Several studies have assessed the relationship between EEG and CBF in acute stroke survivors, finding that perfusion is inversely correlated with measures of EEG delta power (Claassen et al., 2004; Jordan, 2004; Friedman and Claassen, 2010; Finnigan and van Putten, 2013). Furthermore, successful reperfusion by thrombolytic treatment in the acute or subacute stages after stroke was associated with a reduction of delta activity (Finnigan et al., 2006; Finnigan and van Putten, 2013). However, relatively little work has been done on the long-term effects of chronic hypoperfusion and its role in functional impairment at the later stages after stroke, or its relationship with oscillatory activity. The findings reported in the literature suggest that pathological slow-wave activity in chronic stroke may reflect reversible dysfunction related to hypoperfusion. However, more work is needed to investigate this relationship between electrophysiological abnormalities, alteration in blood flow, and cognitive dysfunction. It is not clear if and how reduction in brain signal complexity, and variability relate to compromised perfusion. Source localization of MEG measurements in combination with more sensitive methods of CBF quantification, such as Arterial Spin Labeling (ASL) provides an exciting possibility.

### Present study

In the present study, we characterized the resting-state neural dynamics using two complementary measures of spontaneous neural activity: MEG and fMRI. Resting-state data were acquired from patients with post-stroke aphasia resulting from a single left-hemisphere stroke, from healthy young adults, and from healthy older adults matched in age and education to the stroke patients. The complexity of spontaneous brain activity was quantified from MEG data using spectral and entropy measures, as done in our previous MEG study of stroke patients (Chu et al., 2015). In the present study, we extended previous findings by calculating sample entropy at both short and long time scales, taking advantage of the longer periods of resting data available in this dataset. To estimate BOLD signal temporal variability, we computed two popular measures of BOLD signal variance: standard deviation (SD_BOLD_; Garrett et al., 2011) and mean squared successive difference (MSSD; Mohr and Nagel, 2010; Leo et al., 2012). We also computed the sample entropy (Sokunbi, 2014) of the BOLD signal, to provide a measure more directly comparable to the MEG entropy measure.

The main goal of this study was to assess the utility and sensitivity of signal variability and complexity measures in MEG and fMRI, as indicators of perilesional dysfunction in chronic stroke, and also of age-related changes. Since most neuroimaging studies are conducted in young adults, it is critical to distinguish stroke-related changes from the basic effects of advanced age. Because reductions in complexity and variability have been reported in both stroke and aging with these methods, we conducted a comprehensive study comparing both stroke patients and older controls with younger controls, evaluating the ability of MEG and fMRI to reveal specific changes related to either stroke-induced dysfunction or aging. To our knowledge, no previous studies have investigated the effects of stroke on BOLD signal variability.

In addition to measuring resting-state activity with MEG and fMRI, we also obtained measures of resting blood flow in the same participants. To estimate CBF, we used pulsed Arterial Spin Labeling (pASL). We predicted that hypoperfused perilesional tissue would be more likely to show a reduction in signal complexity and variability.

In summary, the main aim of this study was to characterize changes in the complexity and variability of spontaneous neural signals associated with aging and stroke. In addition, we aimed to characterize the relationship between these measures of brain functional efficiency and abnormalities in blood flow. The combination of complexity, variability, and blood flow measures may help to define the extent of perilesional tissue that is functionally compromised, allowing us to characterize patients' functional lesions more accurately, and to target interventions to ameliorate the dysfunction.

## Materials and methods

### Participants

MEG and MRI data were acquired from three groups of participants: 19 patients with aphasia (3 females); 19 age-, education-, and gender-matched healthy controls; and 24 young controls. Of the young controls, 20 completed resting-state fMRI and 19 completed ASL measurements, and 15 completed resting-state MEG, with an overlap of 11 completing all components. All older controls and patients completed all assessments. This study was approved by the Research Ethics Board at Baycrest Health Sciences. All volunteers gave their written informed consent prior to the study and were compensated for their participation. Individual patient demographic and clinical characteristics are presented in Table 1. Three patients suffered hemorrhagic stroke, and the rest of the stroke cases were ischemic. The most common cause of ischemia was embolism or thromboembolism of cardiac origin.

**Table 1:** **Demographic, clinical, and lesion characteristics for stroke patients**.

**Patient**	**Age (years)**	**Education (years)**	**Handedness^a^**	**Time post-onset**	**Etiology**	**Aphasia type**	**Lesion volume^b^**	**% of left cortex damaged**	**Lesion location**
P1	47	18	Right	4y 1m	Ischemic cause unknown	Nonfluent/agrammatic/ (moderate Broca's)	34,032	5.07	Left post-central, BA47, insula, LSMG, LSTG, LMTG, LITG, L temporal pole
P2	67	21	Right	15y 5m	Ischemic/embolic	Nonfluent (severe Broca's)	169,128	22.53	Left post-central, left pre-central, LSFG, LMFG, BA44, BA45, BA 47, L insula, L STG, L temporal pole, L LMTG, LITG, FUS, LSPL, LSMG, LAG, LBG
P3	70	24	Right	1 y	Ischemic	Mild anomia	4904	Subcortical lesion	Basal ganglia
P4	75	15	Right	2 y 4m	Ischemic/embolic	Conduction	34,440	4.20	Left precuneus, LSPL, LSMG, LAG, LSTG, LMTG, LITG
P5	79	10	Right	2 y 1m	Ischemic/embolic	Mild nonfluent	37,896	4.80	Left post-central, left pre-central, BA44, insula, LSTG, LSPL, LSMG, LAG, precuneus
P6	46	15	Right	2y 3m	Ischemic/thrombo embolic	Mild conduction/ anomia	33,904	4.84	BA 47, left insula, LSTG, LMTG, LITG, left temporal pole, LSMG, LAG, basal ganglia
P7	62	16	Right	1y 2m	Ischemic cause unknown	Conduction/anomia	53,456	6.92	L insula, LSTG, LMTG, LITG, left temporal pole, LFUS, LSMG, LAG
P8	84	19	Right	10y	Ischemic/thrombo embolic	Mild anomia	3176	Subcortical lesion	Basal ganglia
P9	73	19	Left	5y 8m	Hemorrhagic/ICH	Mild anomia	27,440	4.09	LSTG, LMTG, LITG, left temporal pole, LSPL, LSMG, LAG
P10	77	20	Right	7m	Ischemic/thrombo embolic	Severe Wernicke's	23,648	2.81	LSTG, LMTG, LSMG, LAG
P11	66	20	Right	5y 3m	Hemorrhagic	Conduction	78,616	9.59	Left post-central, LSTG, LMTG, LITG, LSPL, LSMG, LAG, precuneus, basal ganglia
P12	58	14	Right	1y 1m	Ischemic/thrombo embolic	Nonfluent, expressive (severe Broca's)	148,904	19.32	Left post-central, Left pre-central, LSFG, LMFG, BA44, BA45, BA47, LSTG, LMTG, left temporal pole, LIFG, L insula, LSMG, LAG, basal ganglia
P13	46	16	Right	4y	Ischemic/embolic	Nonfluent (moderate Broca's)	101,584	12.71	Left post-central, Left pre-central, LSFG, LMFG, BA44, BA45, BA47, L insula, left temporal pole, LSMG, basal ganglia
P14	57	12	Right	2y	Ischemic/embolic	Nonfluent/expressive (severe Broca's)	146,160	20.31	Left post-central, Left pre-central, LSFG, LMFG, BA44, BA45, BA47, L insula, LSPL, LSMG, LAG, LSTG, LMTG, left temporal pole
P15	65	20	Right	6y 1m	Ischemic thrombo embolic	Nonfluent/expressive (severe Broca's)	158,936	21.96	Left post-central, Left pre-central, LSFG, LMFG, BA44, BA45, BA47, L insula, LFUS, LSPL, LSTG, LMTG, LITG, left temporal pole, LSMG, LAG, left precuneus, basal ganglia
P16	68	13	Right	3y 3m	Hemorrhagic/ICH	Mild anomia	22,152	3.24	Left post-central, left precueneus, LSPL, LSMG, LAG
P17	60	14	Right	8y 8m	Ischemic cause unknown	Mild conduction	103,896	14.89	Left pre-central, LSFG, LMFG, BA44, BA45, LSMA, L insula, LSPL, LSMG, LAG, left precuneus, LSTG, LMTG
P18	69	15	Right	1y	Ischemic/thromboembolic	Moderate nonfluent, anomia	9104	0.0013	Left basal ganglia
P19	68	14	Right	4 y 7m	Ischemic cause unknown	Mild anomia	54,192	6.67	Left pre-central, Left insula, LSPL, LSMG, LAG, LMTG
Mean	65.11	16.58		4.26					
SD	10.86	3.56		3.89					

a *Handedness assessed using Edinburgh Handedness Inventory*.

b *Volume of lesioned voxels in microliters*.

*ICH: Intracerebral hemorrhage*.

Participants with aphasia suffered a single left hemisphere stroke at least 6 months prior to the study. They were recruited from several sources in Toronto, Ontario and surrounding areas. These included the stroke clinics at Baycrest and Sunnybrook Health Sciences Centres, the Aphasia Institute (www.aphasia.ca), and Aphasia and Communication Disabilities Program, March of Dimes Canada. Patients ranged in age from 46 to 84 years (Mean = 65.1, SE = 2.49), and had 10–24 years of education (Mean = 16.58, SE = 0.82). All aphasic participants but one were right handed as measured by Edinburgh Handedness Inventory (Oldfield, 1971; Williams, 2010). They were native speakers of English, and had normal hearing and normal or corrected-to-normal vision. All patients retained sufficient capacity of language comprehension to consent for the study and follow task instructions. Exclusion criteria were earlier neurological diseases, language disorders, head traumas or brain surgery, epilepsy, severe psychiatric disorders, and unstable or poor health. Aphasic participants were matched with a group of healthy older controls for gender, age [*t*_(36)_ = 0.155, *p* > 0.05], and education [*t*_(36)_ = 1.02, *p* > 0.05]. Participants were diagnosed with aphasia prior to the study by a speech language pathologist and/or board-certified neurologist. Aphasia diagnosis was determined on the basis of the convergence of the clinical presentation, narrative speech samples, and the results of standardized tests.

Healthy volunteers were recruited from the greater Toronto area by REB-approved advertisements from the University of Toronto community and from the Baycrest Health Sciences subject pool. Both groups of neurologically unimpaired participants were native speakers of English. All young (11 females; age: Mean = 24.63 years, SE = 0.58; education: Mean = 16.88 years, SE = 0.48) and older age-matched controls (3 females, age range: 45–80 years old, Mean = 65.63, SE = 2.31; education range: 12–21 years, Mean = 17.57, SE = 0.53) were right handed and reported normal hearing and normal or corrected-to-normal vision. Participants had no history of neurological, psychiatric, language, or learning disorders and none were taking neuroleptic or mood-altering medications at the time of the study.

### Cognitive and language assessment

Prior to participation in the MEG experiment, patients and age-matched controls completed an extensive neuropsychological battery to assess several domains of cognitive and language functioning. The neuropsychological data were collected as part of a larger study and will be reported fully elsewhere. Age-matched controls participated in all behavioral and neuroimaging assessments completed by the stroke patients, whereas the younger controls only completed the neuroimaging components. All control participants tested within normal limits on all cognitive and linguistic tests. Table 2 presents selected language and cognitive test scores for each patient, and mean scores for the control group.

**Table 2:** **Language test scores for individual patients and control group averages**.

	**BNT**	**PPVT**	**NAVS_SCT**		**NAVS_SPPT**		**Western Aphasia Battery (Bedside form)**						
			**Can**	**Non-Can**	**Can**	**Non-Can**	**PALPA9**	**Flu**	**Comp**	**Rep**	**Obj Nam**	**Aphasia Score**	**Language Score**
P1	8	118	93.33	93.33	20	26.67	NT	5	8	7.5	9.5	78.3	75
P2	NT	93	73.33	40.00	NT	NT	NT	NT	NT	NT	NT	NT	NT
P3	4	100	100	100	100	100	100	10	10	10	10	96.7	95
P4	2	105	100	73.33	66.67	53.33	98.75	8	10	7.5	7.5	80	77.5
P5	17	121	100	100	100	100	NT	8	10	9.5	10	95.8	91.9
P6	5	100	100	73.33	60	33.33	93.75	8	9	7.5	10	90.83	86.87
P7	3	90	80	40.00	NT	NT	NT	6	8	4	8	62	62.5
P8	6	98	100	100.00	86.67	80	NT	9	9	10	9.5	94.17	89.37
P9	8	122	80	66.67	93.33	86.67	NT	9	10	10	10	98.3	97.5
P10	1	0	60	53.33	NT	NT	66.25	7	9	6	6	62	51
P11	9	118	66.67	73.33	40	20	NT	9	10	9	10	97	96
P12	1	93	80	86.67	33.33	6.67	81.25	2	10	5	2	45	38.75
P13	5	101	100	100	100	93.33	98.75	6	10	9.5	10	52.5	70.5
P14	1	74	80	46.67	NT	NT	92.5	2	8	6	7	58.3	48.75
P15	1	87	53.33	80	NT	NT	NT	0	7	3.5	0.5	25	22.5
P16	10	98	100	60	100	100	97.5	9	9	10	10	95	96.25
P17	3	91	100	60	73.33	20	98.75	8	9	7	10	86.7	85
P18	2	94	86.67	86.67	40.00	40.00	88.75	4	9	9	9	75	66.88
P19	8	99	93.33	100.00	93.33	53.33	100	8	10	8.5	10	90.8	86.9
Mean	5.22	94.8	86.66	75.44	71.90	58.09	92.39	6.55	9.17	7.75	8.28	76.86	74.34
SD	4.22	26.1	15.1	21.09	28.67	34.34	10.42	2.87	0.92	2.12	2.85	21.46	22.03
Control Means	10.32	115.74	100	100	100	99.30	97.50						
SD	2.77	13.09				2.1	2.68	N/A	N/A	N/A	N/A	N/A	N/A

*BNT, Boston Naming Test (Kaplan et al., 2001). Scores scaled based on age and years of education; PPVT, Peabody Picture Vocabulary Test (Dunn and Dunn, 2007); NAVS, Northwestern Assessment of Verbs and Sentences (Thompson, 2011); NAV_SCT, Sentence Comprehension Test; Can, Total score on all canonical sentences; Non-Can, Total score on all non-canonical sentences; NAVS_SPPT, Sentence Production Priming Test; PALPA, Psycholinguistic Assessments of Language Processing in Aphasia (Kay et al., 1992); PALPA_word repetition, PALPA 9 total score on word repetition subtest, Western Aphasia Battery- Bedside version (Kertesz, 1982); Flu, Spontaneous Speech Fluency; Comp, Auditory Verbal Comprehension; Rep, Repetition; Obj Nam, Object Naming; NT, Not tested. Bedside Aphasia Score was determined by summing the Speech Content, Fluency, Auditory Verbal Comprehension, Sequential Commands, Repetition, and Object Naming scores, dividing the sum by 6 and then multiplying result by 10*.

*Bedside Language Score was determined by summing the Speech Content, Fluency, Auditory Verbal Comprehension, Sequential Commands, Repetition, Object Naming, Reading, and Writing scores, dividing the sum by 8 and multiplying the result by 10*.

### Resting MEG and fMRI data acquisition

Spontaneous brain activity was recorded while participants looked at a white fixation cross presented in the center of the screen on a black background. During the recording participants were asked to relax and to minimize head and body movements. Resting-state data was collected for 5 min during MEG acquisition and 6 min for BOLD fMRI.

### Structural MRI acquisition and processing

MRI scans were conducted in a single 1 h session, and were always acquired after the MEG session, either the same day or up to 2 weeks after. MRI scans were acquired on a 3-Tesla scanner (Siemens TIM Trio) located at Baycrest. Acquisition included the T1-weighted MPRAGE, fMRI BOLD and ASL scans reported here, as well as T2 FLAIR and diffusion tensor imaging (to be reported elsewhere). A 3D high-resolution T1-weighted anatomical image was used to construct a head model for MEG source modeling (MPRAGE, 1 mm isotropic voxels). MR-visible markers were placed at the fiducial points for accurate registration, aided by digital photographs taken during the MEG session. Processing of anatomical images employed routines from AFNI (afni.nimh.nih.gov/afni) and FSL (http://www.fmrib.ox.ac.uk/research/analysis-group) software packages. T1 images were skull stripped in AFNI.

In stroke patients' MRIs, lesion borders were delineated using segmentation tools in FSL and region of interest (ROI) drawing tools in AFNI, based on a T1 intensity threshold followed by manual adjustment. Regions of gliosis were included in the lesion mask on the basis of the hyperintense signal seen in a coregistered T2-FLAIR image. For spatial normalization into MNI space, we computed a nonlinear warp of each subject's brain to a single-subject template, the “colin27” brain, using the software package ANTS (Avants et al., 2011). Both T1 images and lesion masks where warped into MNI space for group analysis of lesion characteristics and to overlay source-localized MEG images. The same spatial transformation was applied to the results of all functional signal measures computed from fMRI and MEG data, to allow for statistical analysis across participants.

**Figure 1:**
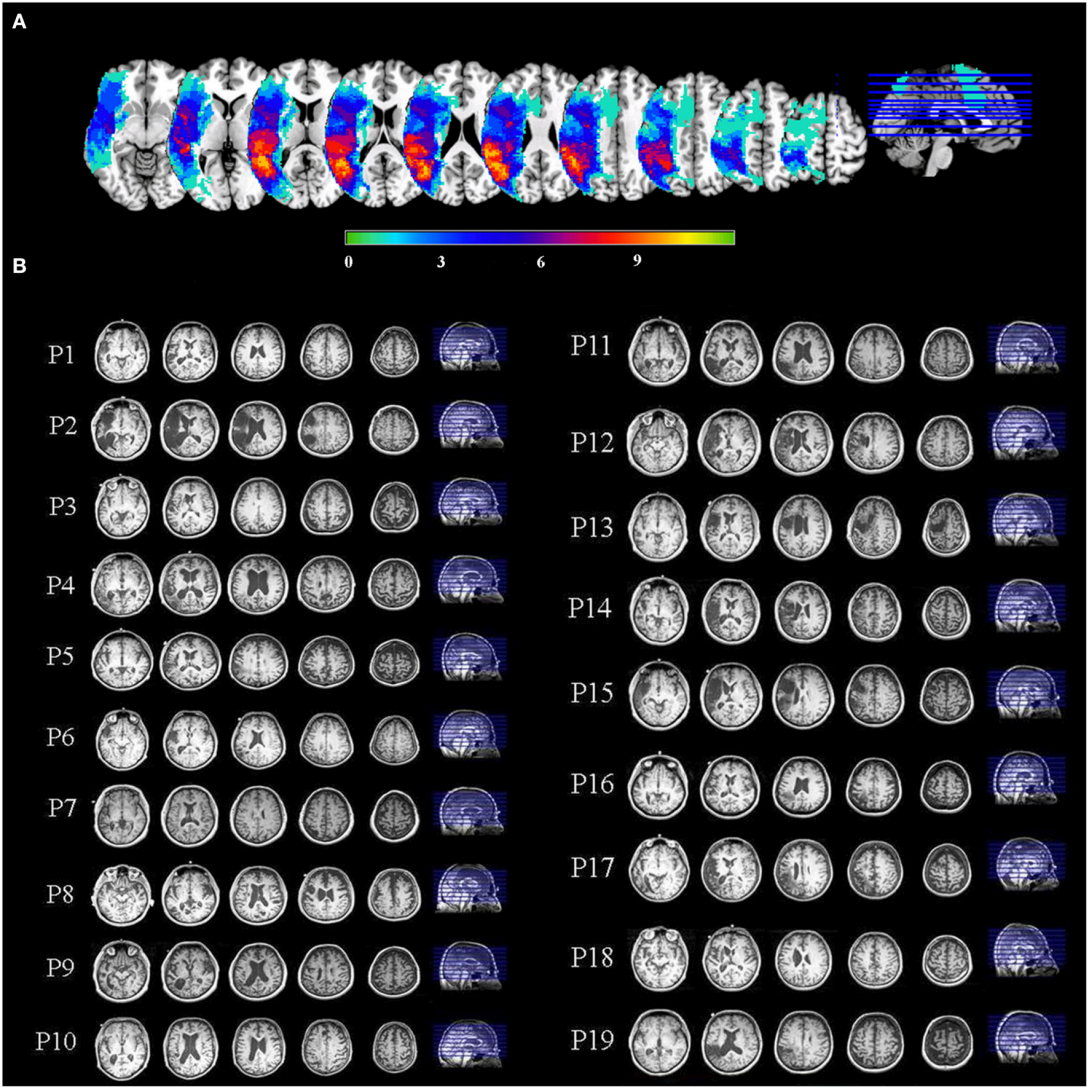
**(A)** An overlay of stroke patients' lesion distributions displayed on a template brain in MNI space. Colors represent the number of patients with a lesion in each voxel. Warmer colors indicate areas of greater lesion overlap. **(B)** Selected slices from T1 images of individual aphasic participants showing lesion sites.

For display of statistical maps derived from patient data, a composite lesion mask was constructed to identify regions that were damaged in the patient group. First, the spatially normalized T1-weighted anatomical images of all patients were averaged together. Next, the lesion mask was overlaid on this image by subtracting a percentage of the signal proportional to the number of patients having a lesion at that voxel. This procedure provides a visual approximation of the “average” pattern of lesion extent across individuals, and provides a suitable anatomical underlay on which to display group-averaged functional imaging data. A lesion overlap map showing the distribution of lesions in the left hemisphere is presented in Figure 1A. Selected slices from T1 images of individual aphasic participants showing lesion sites are shown in Figure 1B.

To characterize physiological data in perilesional and contralesional cortex, we constructed a customized “perilesional rim” ROI in each participant, along with a contralesional control ROI in the unlesioned right hemisphere. To make the perilesional ROI, we dilated each patient's lesion mask by 10 mm in all directions. The lesion mask was then subtracted from the dilated mask, leaving a 10 mm “rim,” and the resulting ROI was further masked with a gray matter segmentation image, eliminating voxels outside the cortex. The resulting perilesional ROI was then downsampled to the resolution of the functional data. For comparison, we also reflected the perilesional ROI onto the undamaged right hemisphere. Thus, the size of the perilesional rim ROI varied across patients according to the lesion size, but the right hemisphere control ROI was the same size as the perilesional ROI for each participant.

### Resting fMRI acquisition and analysis

Resting-state fMRI was acquired using a gradient echo echoplanar imaging sequence with 30 axial oblique slices covering the entire brain (TE = 30 ms, TR = 2 s, voxel size 3.1 × 3.1 × 5 mm^3^, 180 time points, acquisition time: 6 min). The functional data were preprocessed using AFNI. The data were slice-time and motion corrected by registering all functional volumes to the third volume of the run. The functional volumes were coregistered with each subject's structural volume using a 12-parameter affine transformation. Data were smoothed using an 8 mm Gausian kernel. Next, voxel time series were further adjusted by regressing out motion correction parameters and averaged time series extracted from white matter (WM) and cerebrospinal fluid (CSF) masks. For WM and CSF, the time series were extracted from unsmoothed data. The resulting adjusted time series were then normalized so that the overall four-dimensional mean across all in-brain voxels was 100. Following this preprocessing, the measures described below were computed on the resulting time series. The results of these computations were spatially normalized into MNI space.

#### BOLD variability measures

We computed two measures of BOLD variability that have been previously employed in comparisons between clinical groups. One is simply the standard deviation (SD) of the signal (Garrett et al., 2010). Another is MSSD, which emphasizes successive differences in the time series and is therefore more immune to the influence of long-term trends (Leo et al., 2012). MSSD is the mean of the squared differences between each successive time-point and the next time point.

#### BOLD sample entropy (SampEn_BOLD_)

To more directly compare the sensitivity of MEG and fMRI, we also computed Sample Entropy on the resting-state BOLD signal. Sample entropy is identical to the MSE measure used for the MEG data, except limited to only one scale—the TR of the fMRI data. Averaging over successively longer time scales would have yielded insufficient data points for robust calculation of SampEn. Details of the entropy calculations are given in the MEG analysis section. Parameter values for SampEn_BOLD_ were the same as for MEG: *m* = 2, *r* = 0.2.

### MEG acquisition and processing

MEG signals were recorded with a 151-channel whole-head system with axial gradiometers (VSMMedTech, Coquitlam, Canada). MEG was recorded continuously at a sampling rate of 625 Hz, and acquired with online synthetic 3rd-order gradient noise reduction (Vrba and Robinson, 2001). In addition to the resting-state measurements, participants completed a language processing experiment in the MEG scanner, which will be reported separately. The 5-min continuous resting data set was arbitrarily divided into epochs of 5 s. Head position with respect to the MEG helmet was monitored using three coils placed at anatomical landmarks of the head (nasion, left, and right pre-auricular points). The head position was measured at the beginning and end of the resting-state run, and the average of the two measurements was used for source analysis.

To construct head models for MEG analysis, the locations of the fiducial points were marked manually in AFNI software (Cox, 1996), and the T1-weighted MRI was spatially transformed into the coordinate space of the MEG data. The skull was stripped in AFNI, and a 3D convex hull approximating the inner surface of the skull was constructed using the software package Brainhull (http://kurage.nimh.nih.gov/meglab/Meg/Brainhull). Taking into account the position of the head relative to the sensors, a multi-sphere head model (Huang et al., 1999) was computed for each MEG session, with the spheres tangent to the inner surface of the skull. All maps of MEG signal parameters were computed originally in the MEG-based coordinate system, and then warped into MNI space for statistical analysis across participants.

### MEG data analysis

Raw MEG sensor signals were screened for artifacts, and epochs containing obvious signal disruptions were rejected (<1% of all epochs). Signals were low-pass filtered at 80 Hz and downsampled to 208.33 Hz prior to beamforming analysis. All further signal analysis was conducted in source space using Synthetic Aperture Magnetometry (SAM) beamforming. Analysis of “virtual channel” signals in source space has two advantages (beyond localization) compared to analysis of sensor data: (1) the beamforming procedure attenuates extracranial artifacts such as blinks, eye movements, and muscle activity (Vrba, 2002; Cheyne et al., 2007), and (2) source-space analysis compensates for differences in head shape and head position across participants, which strongly affect the propagation of electromagnetic activity from the brain to the sensors, which are fixed in the MEG helmet. Note that we did not reject trials based on blinks because the beamforming procedure effectively removes them from the virtual signals estimated for intracranial locations, with the possible exception of signals in the orbitofrontal cortex adjacent to the eye orbits (Bardouille et al., 2006). The remaining artifacts were caused by disturbances arising from environmental noise and subject motion.

### Resting-state MEG analysis

MEG source analysis was conducted using SAM (Vrba and Robinson, 2001), as implemented in CTF software (CTF; Port Coquitlam, British Columbia, Canada). SAM is a beamformer technique that can be used to compute the full time course of virtual channels at selected individual locations, or on a regular grid of locations (voxels) spread across the brain. SAM is a scalar beamformer, in which a nonlinear optimization technique is used to select one direction of current flow at each voxel to maximize dipole power. In short, SAM provides a series of sensor weights for each voxel; the weights are computed so as to pass signal from a dipole located in the target voxel, while minimizing signal power from all other locations. We computed weights on a whole-brain grid of locations spaced 1 cm apart. These weights were then multiplied with the original sensor time series data to yield a new, spatially filtered, time series signal at each voxel (1 cm^3^). Normalized weights were used to render virtual signals in dimensionless units of signal-to-noise ratio, with noise power estimated as the lowest singular value of the sensor covariance matrix (Vrba and Robinson, 2001). Signals were filtered at 0–80 Hz prior to beamforming. Power spectra of spontaneous signals at each voxel were estimated using Welch's method in Matlab (Fast Fourier Transform of 500 ms Hamming windows with 50% overlap). Power spectra were averaged across epochs.

To evaluate quantitative parameters related to slowing of spontaneous activity, we computed measures of relative power from the resulting power spectra. Relative power of the delta (1–4 Hz), theta (4–7 Hz), alpha (8–12 Hz), and beta (15–30 Hz) frequency bands were calculated as the ratio of the power of each specific frequency band divided by the total power across 0–80 Hz. Computing relative power over the frequency spectrum avoids potential confounds introduced by the normalized beamformer weights (Luckhoo et al., 2014) and effectively bases the analysis on spectral shape rather than levels of absolute power.

MSE was calculated from the time domain virtual signal at each voxel and averaged across epochs (Costa et al., 2005; Park et al., 2007). MSE provides a measure of the complexity of biological signals, while accounting for the multiple time scales inherent in such time series. It is usually defined as the sample entropy of the original time series calculated across various time scales, denoted by a scaling parameter. Sample entropy is the negative natural logarithm of the conditional probability that the two sequences of *m* consecutive data points, with a tolerance = *r*, will remain similar following the addition of the next consecutive data point. Periodic or uncorrelated random signal (e.g., white noise) is low in sample entropy, whereas complex irregular signals are high in sample entropy. Reduced MSE values have been considered a marker of dysfunction in variety of biological systems, including heart rate and DNA sequences (Costa et al., 2005). Reduced MSE has been also demonstrated in brain injury (Beharelle et al., 2012) and Alzheimer's disease (Park et al., 2007; Poza et al., 2007).

Most relevant for the present study, the scaling parameter allows MSE to measure changes in signal complexity at different time scales. In the present study the sample entropy was calculated with the parameters *m* = 2 and *r* = 0.2 from scales 1–5, which corresponds to time steps of 4.8–24 ms. In our previous study with a different cohort of stroke patients (Chu et al., 2015), we observed that perilesional tissue exhibiting increased slow-wave activity also tended to have decreased MSE at similar scales. In addition, in the present study we calculated sample entropy at longer scales (MSE scales 7–20, corresponding to 33–96 ms) to better understand the changes associated with stroke lesions and aging across different time scales.

For each relative power measure, and the MSE average for short (scales 1–5) and long (7–20) time scales, we generated whole-brain 3D maps and spatially normalized them to MNI space.

### ASL data acquisition and processing

CBF measurements were made using a PICORE Q2TIPS pulsed ASL (pASL) sequence (Luh et al., 1999). The label thickness was 110 mm, with a 29 mm gap between the label and imaging regions. The Q2TIPS saturation pulse was applied to the proximal boundary of the labeling slab. Flow crusher gradients were applied with a threshold of 100 cm/s. Other imaging parameters were: 64 × 64 in-plane matrix, 26 slices and 3.5 × 3.5 × 5 mm voxels, with a 0.25 mm gap between slices. The ASL acquisition consisted of 150 frames (75 tag and 75 control), with TI1 = 700 ms, TIs = 1600 ms and TI2 = 1800 ms, chosen to accommodate a wide range of flow rates. The scans used a repetition time (TR) of 3 s, and an echo-time (TE) of 13 ms resulting from GRAPPA echo-planar imaging (EPI) readout with an acceleration factor of 2. A 2D gradient-echo EPI scan (with TR set to 10 s) was used to estimate the equilibrium magnetization of arterial blood.

The raw ASL time series were motion corrected and coregistered to the anatomical MPRAGE image using SPM8. Images were smoothed with a 6 mm FWHM Gaussian blur. To minimize BOLD-contamination, the control-tag difference images were calculated using surround subtraction (Lu et al., 2006), and difference images were averaged across timepoints. CBF was quantified using the one-compartment Standard Kinetic Model (Buxton et al., 1998), implemented in ASLtbx (Wang et al., 2008). Typical parameter values for gray matter CBF (defaults in ASLtbx) were assumed based on prior literature (Wong et al., 1997; Çavuşoǧlu et al., 2009). Quantitative CBF maps were warped into MNI space using ANTS software, with the same nonlinear warp used for the anatomical MPRAGE images.

### Statistical analysis

Whole-brain maps of quantities described above were compared between groups using voxel-wise independent sample *t*-test implemented in AFNI. All statistical results were thresholded at a voxelwise threshold of *p* < 0.01 uncorrected. Additionally, a minimum cluster size of 20 voxels was used for display. To control for multiple comparisons across voxels, we computed the false discovery rate (FDR; Genovese et al., 2002) for each map at the chosen threshold. FDR is an estimate of the upper bound (q) for the proportion of colored voxels that are false positives, and is dependent on the amount of true signal present in the data. *q*-values are indicated for each map in the figures.

## Results

### BOLD signal variability (SD_BOLD_): Effects of aging and stroke

**Figure 2:**
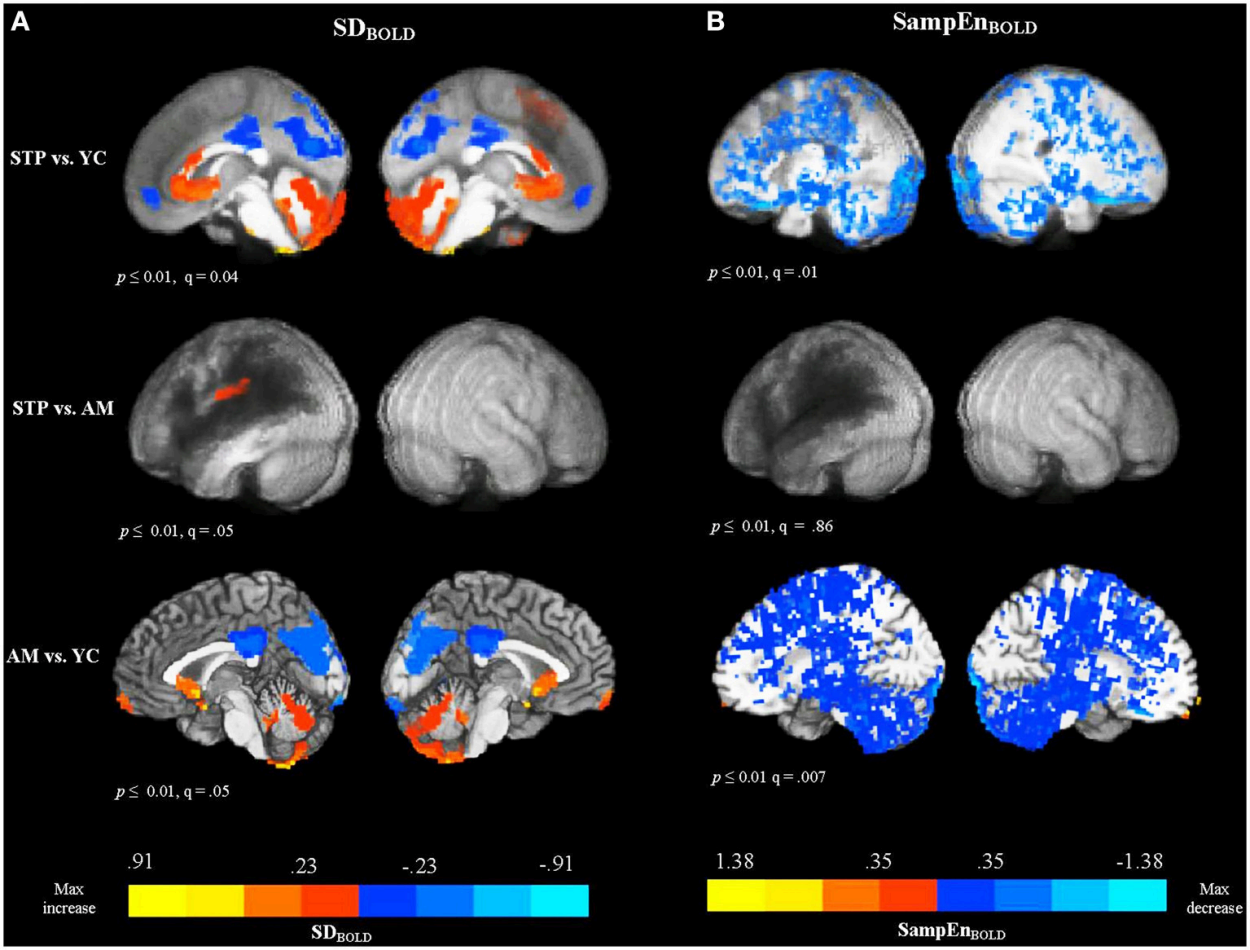
**Between group voxel-wise contrast maps of SD_**BOLD**_ and SampEn_**BOLD**_ values (STP, stroke; YC, young controls; AM, age-matched controls)**. For comparisons with stroke patients the results were overlaid on top of an artificially darkened anatomical image representing the lesion distribution across patients. Darker colors represent greater lesion overlap in these areas. The maps represent the voxel-wise contrast resulting from a *t*-test. The statistical maps were thresholded at a voxelwise threshold of *p* < 0.01 with a minimum cluster size of 20 voxels. False discovery rates (*q*-value) are indicated for each map. The same anatomical underlay and thresholding are used in subsequent figures. Blue colors reflect the decrease in BOLD SDs and SampEn, and red colors reflect increases present in significant voxel clusters within each activation map. **(A)** Group comparison maps of SD_BOLD_ values. **(B)** Group comparison maps of SampEn_BOLD_ values.

One of the stroke patients did not complete the resting-state MRI protocol due to claustrophobia, but did complete the anatomical scan before requesting to stop, allowing for their inclusion in the MEG analysis. In total, resting MRI data was available for 18 stroke patients, 19 age-matched controls, and 20 young controls. Figure 2A shows voxel-wise group comparison maps of SD_BOLD_ values. In all group-comparison brain maps in this paper, but not for correlation analyses, the color scale represents mean differences in the quantity of interest (e.g., SD_BOLD_ in this case), while the thresholding is based on *p*-values resulting from a 2-sample *t*-test between groups. The locations of significant clusters are listed in the Supplementary Materials in Table S1. For comparisons with stroke patients, the results are overlaid on top of a darkened rendering representing the lesion distribution across patients. Darker regions represent greater lesion overlap among stroke participants.

The comparison of SD_BOLD_ values for stroke patients with young controls revealed decreased variability for patients primarily in default mode regions, including the left and right anterior medial frontal cortex, posterior cingulate gyrus, precuneus, cuneus, and a smaller cluster in the left lateral parietal cortex (angular and supramarginal gyri). The analysis also revealed areas of increased variability for the stroke patients in the superior and medial frontal gyri, anterior cingulate gyrus, fusiform gyrus, and cerebellum, bilaterally. However, the comparison between age-matched controls and stroke patients did not detect significant decreases in variability in the default mode regions, indicating that patients and older age-matched controls exhibited equivalent variability of the resting-state BOLD signal in these areas. There was one cluster of significant increase for stroke patients observed in the left postcentral gyrus, extending to the inferior parietal cortex. The comparison of SD_BOLD_ maps for age-matched controls with young controls revealed decreased BOLD signal variability for older participants mainly in the default mode regions [i.e., posterior cingulate gyrus, precuneus, cuneus, and lateral parietal regions (angular and supramarginal gyri, bilaterally)]. The decrease was also observed in the left inferior frontal gyrus, left middle frontal gyrus, left middle temporal, and middle occipital gyrus, bilaterally. The analysis also revealed areas of increased variability for the older adults in the left and right medial frontal gyri and superior frontal gyri, as well as anterior cingulate gyrus, left fusiform gyrus, and cerebellum, bilaterally. MSSD_BOLD_ values showed a very similar pattern of results. The group comparison maps of MSSD_BOLD_ values are shown in Supplementary Materials (Figure S1 and Table S2).

### BOLD signal sample entropy (SampEn_BOLD_): Effect of aging and stroke

The comparison between patients and young controls using a voxel-wise *t*-test, revealed that patients exhibited significantly reduced SampEn_BOLD_, with a widespread distribution (see Figure 2B). Because of the broadly distributed effects that this measure produced we did not list individual regions in the tables. Convergent with the variability measures, there were no significant differences in the SampEn_BOLD_ values between patients and age-matched controls, indicating that patients and age-matched controls did not differ in the complexity of resting BOLD activity. Compared to young controls, older participants showed reduced SampEn of the resting BOLD signal, with a widespread bilateral distribution. In summary, all measures of resting-state BOLD variability and complexity revealed significant changes in older participants compared to younger participants, regardless of stroke status. Stroke patients did not differ significantly from age-matched controls.

### MEG resting data MSE (scales 1–5): Effects of aging and stroke

**Figure 3:**
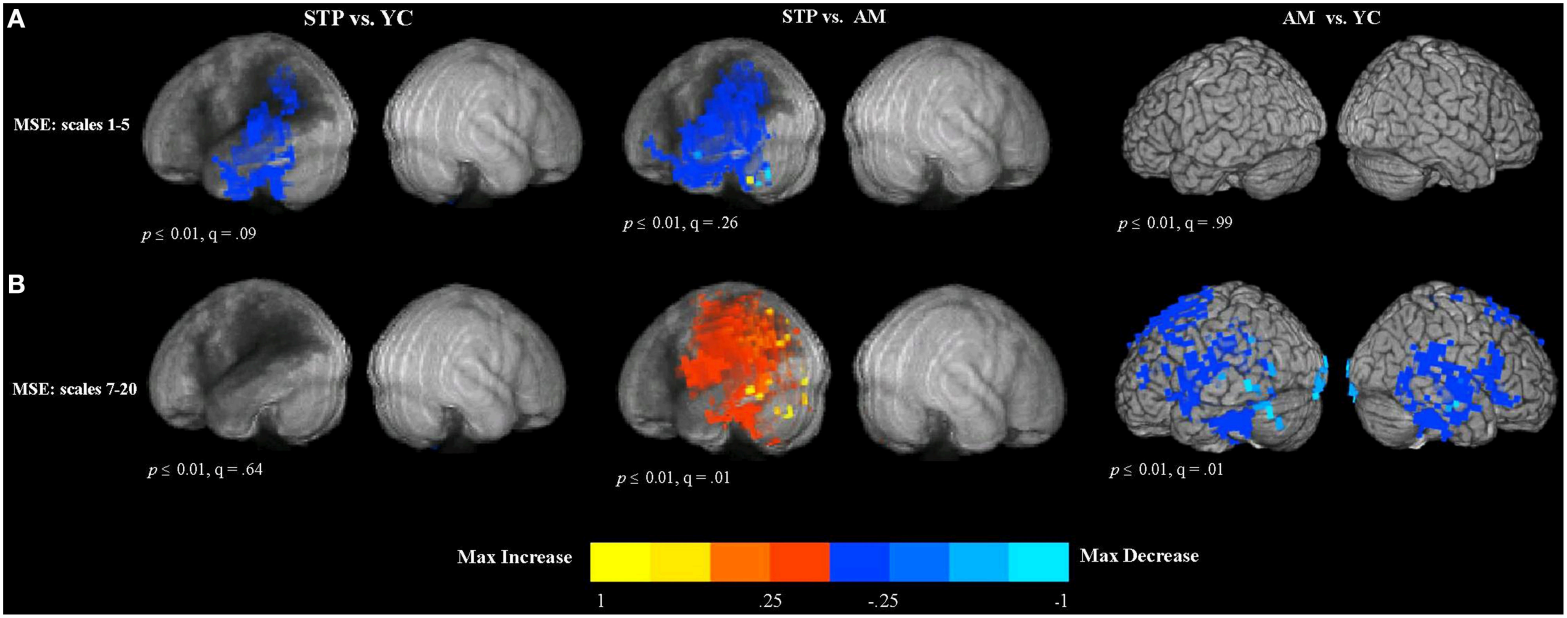
**Between group voxel-wise contrast maps of MSE (STP, stroke; YC, young controls; AM, age-matched). (A)** The between group *t*-test comparison maps of MSE scales 1–5. **(B)** The between group *t*-test comparison maps of MSE scales 7–20.

Resting MEG data was available for 19 stroke patients, 19 matched controls, and 15 young controls. The group comparison maps of MSE at short time scales are presented in Figure 3A. For MEG resting measures, we did not list individual regions in the tables because these effects had a widespread distribution. The maps represent the voxel-wise contrast resulting from a *t*-test. Compared to young controls, patients exhibited significantly lower MSE at the short time scales (1–5) along the left temporal and parietal regions surrounding the region of maximal lesion overlap. The analysis was significant at a voxel-wise threshold *p* < 0.01 with a map-wise FDR of *q* = 0.09. The comparison with age-matched controls revealed a similar pattern of reduced MSE in patients that extended into the left inferior frontal cortex, but with a map-wise FDR of *q* = 0.26, indicating lesser reliability of individual voxels. When these analyses were thresholded at *p* < 0.001 uncorrected, similar activation patterns were present, but with smaller clusters (Figure S2A). There were no significant voxels in the right, unaffected hemisphere in the comparisons between patients and both control groups. No significant differences in short time scale MSE were found between older age-matched and young control groups.

### MEG resting data MSE (scales 7–20): Effects of aging and stroke

The results of group comparisons at the longer time scales are shown in Figure 3B (and Figure S2B at a higher threshold of *p* < 0.001 uncorrrected). The maps represent the voxel-wise contrast resulting from a *t*-test. The analysis was significant at a voxel-wise threshold *p* < 0.01, with the map-wise FDR *q* = 0.01. The comparison with age-matched controls revealed *increased* entropy values for patients that were wide spread in the left hemisphere, including the posterior part of the inferior frontal gyrus, superior and inferior parietal regions, as well as superior, middle and inferior temporal cortex affected by the lesion. There were no significant voxels in the right, unaffected hemisphere in the comparisons between patients and both control groups. There were no significant differences between patients and young controls.

In contrast, the comparison of older age-matched controls with young controls revealed *decreased* MSE at the longer time scales for older adults (see Figure 3B). This decrease was found bilaterally in the superior and dorso-lateral frontal cortex, medial frontal, and middle cingulate cortex, including temporal and parietal regions and also precentral and postcentral cortex along the central sulcus.

### Spectral measures of MEG resting data: Effect of aging and stroke

**Figure 4:**
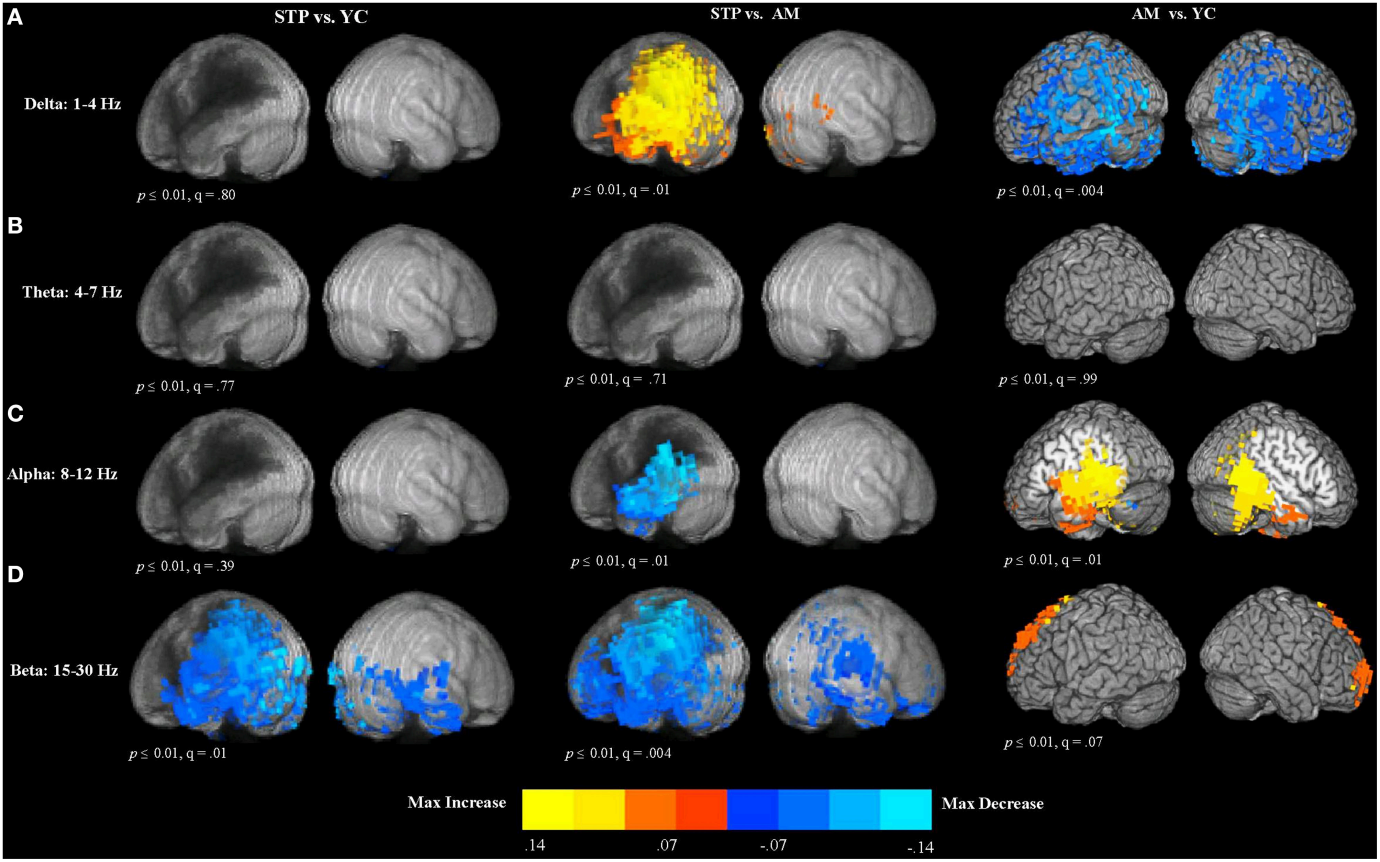
**Between group voxel-wise contrast maps of relative power (STP, stroke; YC, young controls; AM, age-matched). (A)** *T*-test comparison maps of relative delta power. **(B)** *T*-test comparison maps of relative theta power. **(C)** *T*-test comparison maps of relative alpha power. **(D)** *T*-test comparison maps of relative beta power.

To evaluate quantitative parameters related to slowing of spontaneous activity, we computed measures of relative power in the delta (1–4 Hz), theta (4–7 Hz), alpha (8–12 Hz), and beta (15–30 Hz) frequency bands. Relative power was calculated as the ratio of the power of each specific frequency band divided by the total power across 0–80 Hz. The results of between group voxel-wise *t*-tests comparing relative power between patients and control groups are presented in Figure 4.

#### Delta (1–4 Hz)

In comparison to age-matched controls, patients exhibited significantly increased delta power in the left hemisphere regions adjacent to the lesion zone (see Figure 4A). The comparison of older age-matched controls with young controls, revealed significantly *diminished* delta power for older adults. These decreases had a widespread bilateral distribution and included almost the entire temporal and parietal regions, extending into the superior and inferior frontal cortex.

#### Theta (4–7 Hz)

There were no significant differences in theta power between patients and young controls, patients and age-matched controls, and between older and younger control groups (see Figure 4B).

#### Alpha (8–12 Hz)

The comparison of patients with age-matched controls revealed significantly decreased alpha oscillatory activity for patients in the temporal and parietal regions adjacent to the regions of maximal lesion overlap (see Figure 4C). In comparison to young controls, older adults exhibited alpha power *increases* in the left posterior inferior frontal gyrus, precentral gyrus, left insula, middle frontal gyrus, and left medial frontal cortex. Alpha power increases extended into the bilateral temporal and parietal regions.

#### Beta (15–30 Hz)

Compared to young controls, patients showed widespread bilateral beta power decreases that were most pronounced in the left hemisphere areas adjacent to the lesion (see Figure 4D). Beta power decreases were found in the left superior and inferior parietal cortex and also extended over the entire temporal lobe, and into inferior frontal gyrus, precentral cortex, and middle frontal regions. In the right hemisphere, power decreases were also observed in the temporal lobe. When compared to age-matched controls, patients exhibited significantly reduced beta power in the lesioned left hemisphere, spanning the left temporal and parietal lobes, and extending into the precentral and postcentral gyri, superior and middle frontal gyri, anterior frontal and inferior frontal cortex. In the right hemisphere, power decreases were found in the precentral and postcentral gyri, temporal and parietal cortex, and anterior frontal areas. Comparison of older age-matched controls with young controls, revealed beta power *increases* for older adults bilaterally along the superior and middle frontal cortex, extending into the anterior medial frontal cortex.

**Figure 5:**
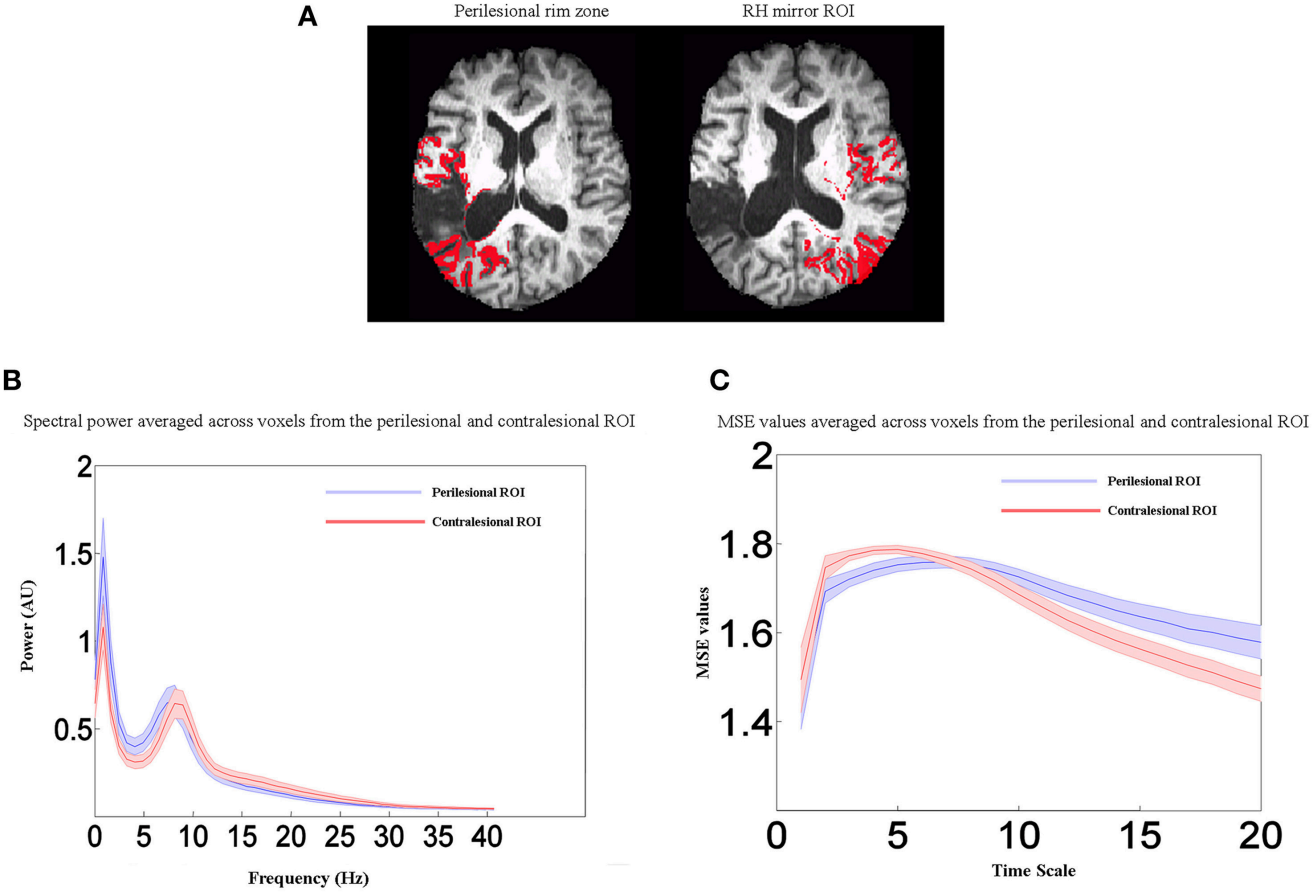
**Perilesional ROI analysis. (A)** Example of perilesional and contralesional ROIs from one participant. **(B)** Averaged power spectra for all stroke patients (*n* = 19) from the perilesional rim ROI and the contralesional control ROI in the unlesioned right hemisphere. **(C)** Averaged MSE values for all stroke patients (*n* = 19) from the perilesional rim ROI and the contralesional control ROI in the unlesioned right hemisphere. Shaded regions represent the standard error of the mean power estimate. Relative to the healthy right hemisphere, perilesional tissue showed a shift toward slower frequencies (delta and theta bands), decreased entropy at lower time scales from 1 to 5, and increased entropy at longer time scales. Scales 1, 2, 3 etc. represent time steps of 4.8, 9.6, 14.4 ms etc.

In summary, our results show that, in comparison to control groups, stroke patients exhibited changes in electrophysiological activity in the areas surrounding the lesion. To further illustrate the frequency- and scale-dependent nature of the observed changes, we computed averaged power spectra and MSE curves from the perilesional rim ROI and the contralesional control ROI in the unlesioned right hemisphere. Example perilesional ROIs from one participant are shown in Figure 5A, but the data shown here are averaged across all 19 stroke patients. Figure 5B displays averaged power spectra in the two ROIs. Relative to the healthy right hemisphere, perilesional tissue showed a shift toward slower frequencies, with increased power in the delta and theta bands up to about 8 Hz, but decreased power in the upper alpha and beta bands, up to about 30 Hz. MSE at different time scales are shown in Figure 5C. Note that the time scales shown are dependent on the sampling rate of the data as analyzed (here 208.33 Hz), so that scales 1, 2, 3 etc. represent time steps of 4.8, 9.6, 14.4 ms etc. Perilesional tissue exhibits decreased entropy at lower time scales from 1 to 5, corresponding to faster changes in signal values and therefore to higher frequencies. Perilesional tissue also exhibits increased entropy at longer time scales, corresponding to slower fluctuations and hence lower frequencies. Thus, analyses of spectral power and MSE converge on similar conclusions: perilesional tissue exhibits an overall slowing of physiological activity, with greater power and signal complexity at longer time scales and reductions in these quantities at finer time scales. (See below for formal analysis of correlations between MSE and spectral measures). Changes in MSE at longer scales also distinguished older from younger controls, but in the opposite direction: older controls had reduced MSE at scales 7–20 relative to young controls, but stroke patients had an increase. In contrast, MSE at the shorter scales (1–5) was specifically sensitive to stroke and not aging, with consistent reductions in stroke patients.

### Cerebral blood flow: Effects of aging and stroke

**Figure 6:**
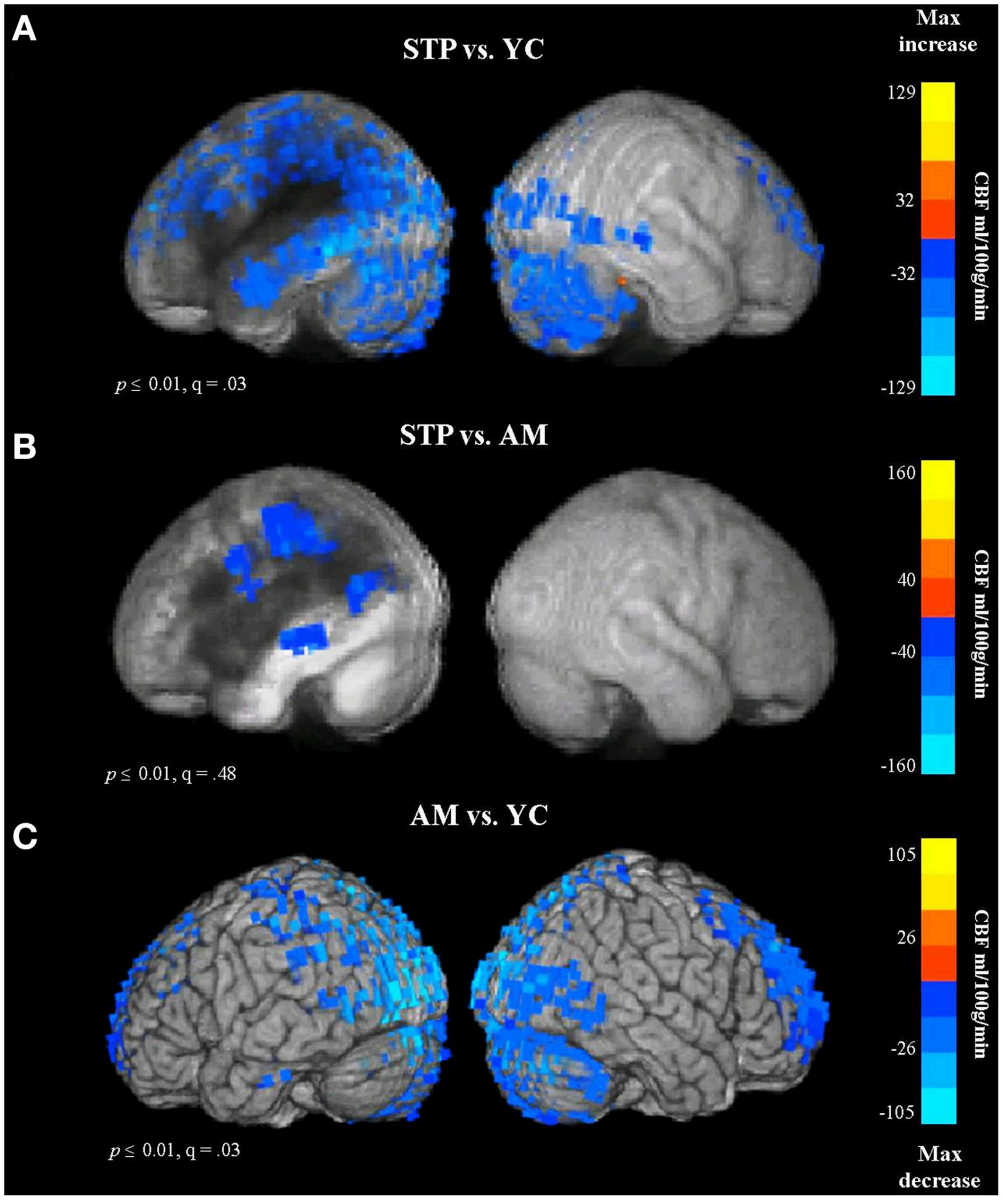
**Group comparison CBF maps obtained with pulsed Arterial Spin Labeling MRI (STP, stroke; YC, young controls; AM, age-matched)**. Blue colors reflect a decrease in CBF. **(A)** CBF for Stroke patients vs. Young controls. **(B)** Stroke patients vs. Older Age matched controls. **(C)** Older controls vs. Young Controls.

In total, CBF measurements were available for 17 stroke patients, 19 age-matched and 19 young control participants. The results of between group voxel-wise *t*-tests comparing CBF values are presented in Figure 6 and Table S3 (in Supplementary Materials). The mean CBF values across the entire gray matter volume were 56 ml/100 g/min in young controls, 39 ml/100 g/min in older controls, and 37 ml/100 g/min in stroke patients.

In comparison to young controls, patients exhibited significantly decreased CBF values in the regions surrounding the lesion. The blood flow decreases spanned the entire left temporal lobe, and included superior and inferior parietal cortex, superior and middle frontal regions, and occipital cortex (see Figure 6A, Figure S3A). When compared to age-matched controls, patients showed reduced blood flow values in the left parietal region, along the left middle temporal gyrus, and extending into the precentral gyrus (Figure 6B). This comparison was significant at voxel-wise threshold *p* < 0.01, but only achieved a maximum FDR of *q* = 0.48, indicating that the significance of individual voxels within the cluster is somewhat unreliable. When thresholded at *p* < 0.001 uncorrected, one cluster remained significant, in the parietal lobe at the superior edge of the lesion zone (Figure S3B). However, the comparison between older age-matched controls and young controls revealed highly significant (*q* = 0.03) reduced blood flow values for older controls along the occipital cortex, superior parietal regions, superior frontal areas, and anterior prefrontal cortex, bilaterally (Figure 6C, Figure S3C), a very different distribution from the apparently perilesional changes seen in stroke-patients vs. age-matched controls (Figure 6B, Figure S3B).

These results indicate that the CBF changes associated with normal aging can be dissociated from stroke related dysfunction. The age-related decline in CBF values was found in the occipital and superior brain regions bilaterally, whereas stroke related reductions in blood flow were specific to the left hemisphere regions affected by the lesion.

### Relationship between cerebral blood flow, BOLD time-series, and MEG signal

To identify the relationship between CBF and the variability and complexity measures obtained from resting MEG and MRI data, we examined correlations between corresponding voxel values across all patients for whom data was available for each analysis, at each voxel in the brain. Spearman's rank-order nonparametric correlation test was used for this analysis to account for possible nonlinearity or nonnormality of the imaging data.

**Figure 7:**
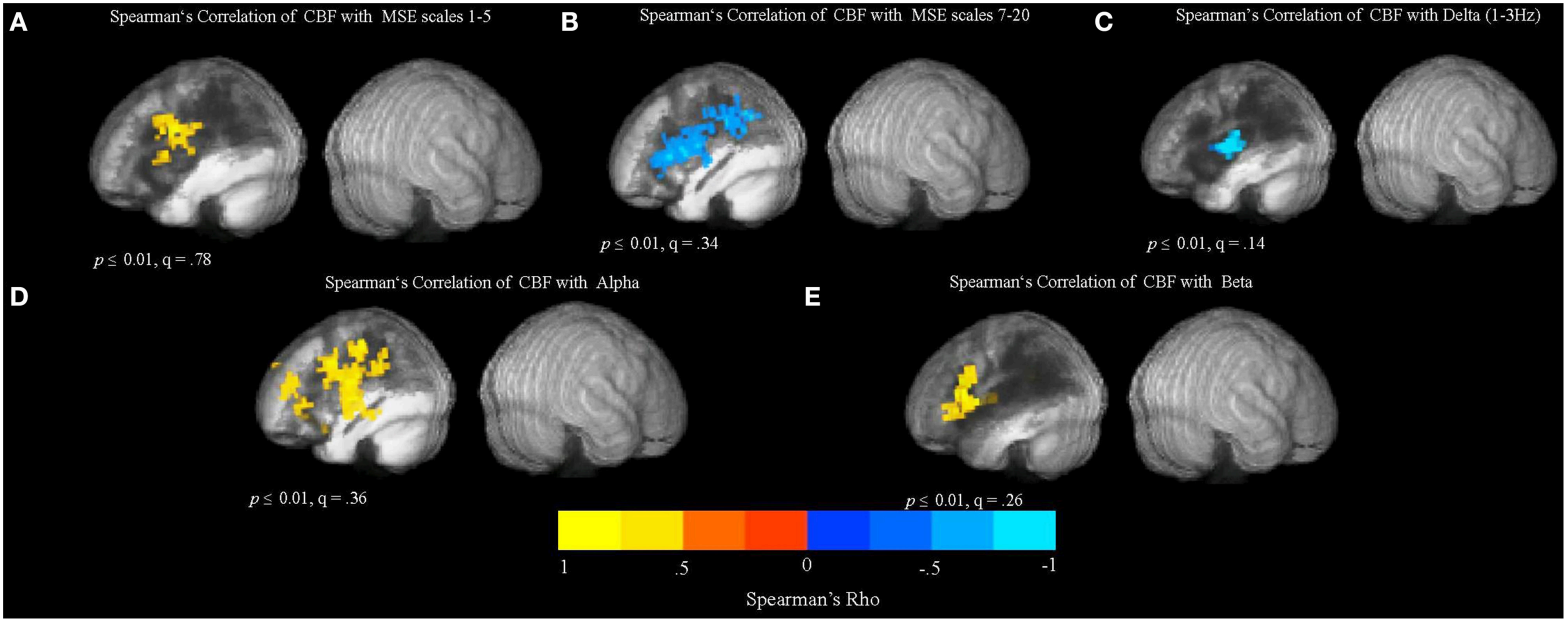
**Relationship between cerebral blood flow (CBF) and the MSE and spectral measures obtained from resting MEG data**. The maps show whole-brain, voxel-wise Spearman's rank-order correlations between CBF and MEG measures (MSE scales 1–5, MSE scales 7–20, and relative spectral band power). **(A)** Spearman's rank-order correlation map between CBF and MSE scales 1–5. **(B)** Spearman's rank-order correlation map between CBF and MSE scales 7–20. **(C)** Spearman's rank-order correlation map between CBF and relative delta power. **(D)** Spearman's rank-order correlation map between CBF and relative alpha power. **(E)** Spearman's rank-order correlation map between CBF and relative beta power.

The resulting Spearman's rank-order correlation maps are presented in Figure 7. The locations of significant clusters are listed in the Supplementary Materials in Table S4. The maps show voxel-wise correlations between CBF and MEG measures (MSE scales 1–5, MSE scales 7–20, relative spectral band power). The correlations were significant at voxelwise threshold of *p* < 0.01, but the map-wise FDR values ranged from *q* = 0.14 to *q* = 0.78, making the localization value of these results more questionable. However, another analysis based on *a priori* perilesional ROIs also revealed significant changes that were identical in directionality (see Section Relationship between CBF and MEG Signal in the Perilesional Rim below). Furthermore, thresholding the same maps at *p* < 0.001 uncorrected produced similar but smaller clusters in the same locations (Figure S4). The analysis revealed a significant positive relationship between CBF values and MSE at time scales 1–5 in the LH regions surrounding the lesion (see Figure 7A), indicating that reduced blood flow in these areas is associated with reduced entropy values at short time scales.

In addition, there was a significant negative correlation between CBF and MSE at longer time scales (7–20), as well as a negative relationship between CBF values and the relative delta power in the LH regions affected by the lesion (see Figures 7B,C). These results suggest that spectral “slowing” was associated with reduced blood flow. In addition, there was a positive relationship between CBF and alpha and beta power (Figures 7D,E). However, there were no significant correlations between CBF and SD_BOLD_ and SampEn_BOLD_ (figure not shown), and there was no significant relationship present in the unaffected RH.

### Relationship between cerebral blood flow and MEG signal in the perilesional rim

The whole brain voxel-wise correlation analysis demonstrated a relationship between reduced blood flow and MEG signal abnormalities, but not resting BOLD measures in stroke patients. However, these analyses were potentially affected by variability in the lesion size and extent. To more directly test the relationship between blood flow and electrophysiological abnormalities, we computed Pearson's correlations on the values extracted from the perilesional rim. This was consistently the region that bordered the lesion in each patient. This perilesional ROI was masked by the lesion map and cortical segmentation in each patient in order to exclude lesioned voxels, white matter, and CSF. For comparison, the same values were extracted from the contralateral mirror right hemisphere ROI for all patients. See Figure 5A for example ROIs from one patient.

**Figure 8:**
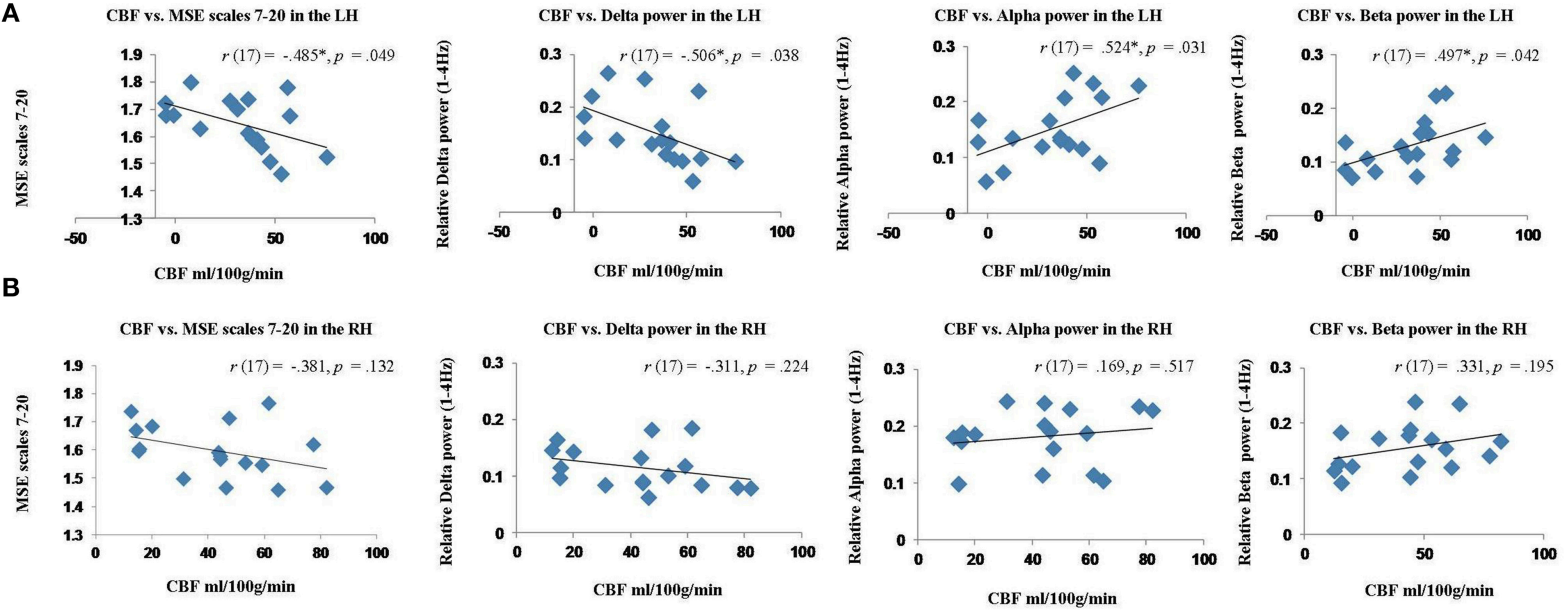
**Scatter plots showing the relationship between cerebral blood flow (CBF) and MEG measures (MSE values and spectral measures) extracted from the perilesional rim and contralateral RH ROIs for stroke patients. (A)** Relationship between CBF and MSE scales 7–20, relative delta, alpha and beta power in the perilesional rim. **(B)** Relationship between CBF and MSE scales 7–20, relative delta, alpha and beta power in the contralesional RH ROI.

**Figure 9:**
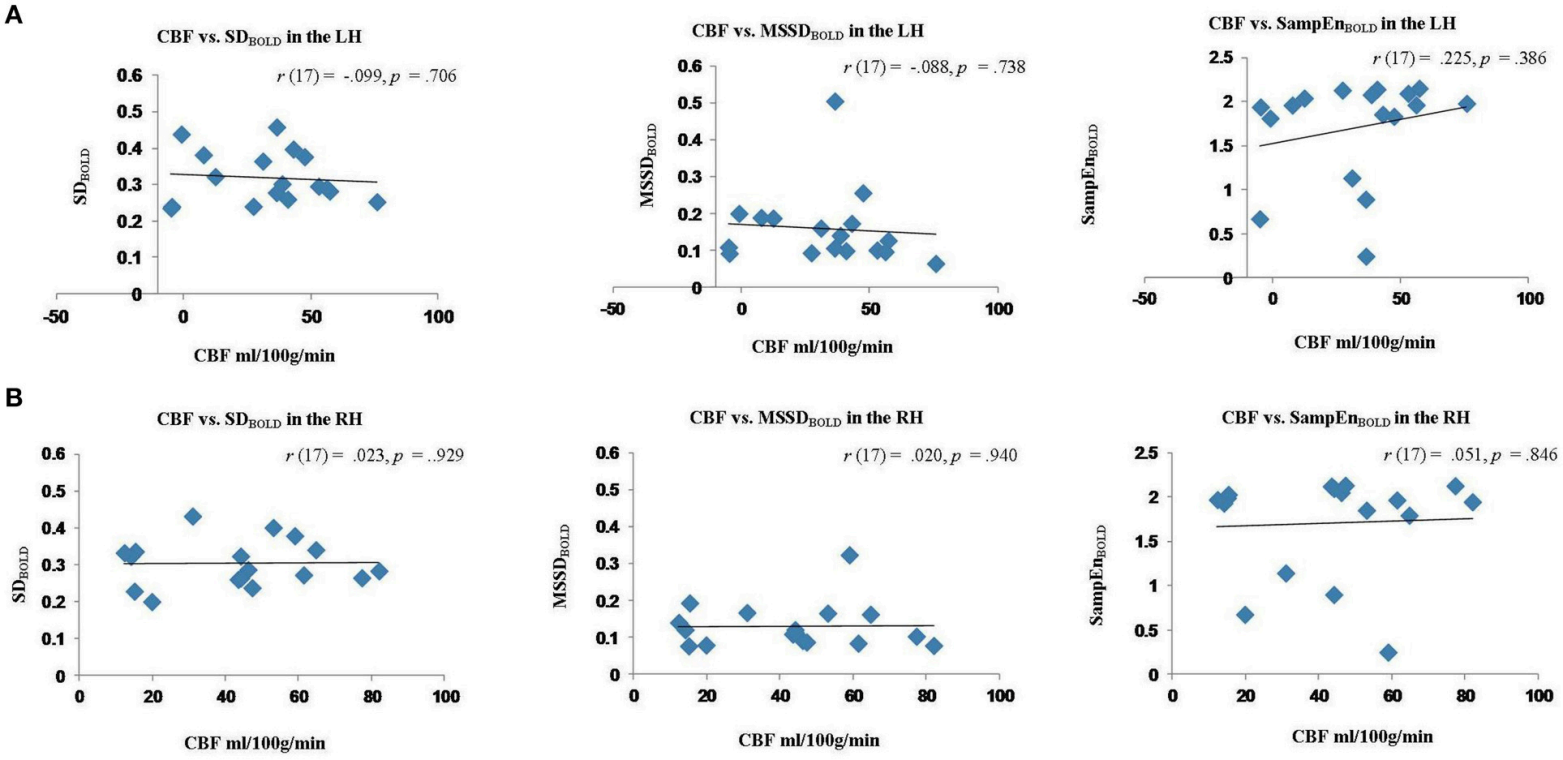
**Scatter plots showing the relationship between cerebral blood flow (CBF) measures of BOLD signal variability and complexity (SD_**BOLD**_, MSSD_**BOLD**_, SampEn_**BOLD**_) extracted from the perilesional rim and contralateral RH ROIs for stroke patients. (A)** Relationship between CBF and SD_BOLD,_MSSD_BOLD,_SampEn_BOLD_ in the perilesional rim. **(B)** Relationship between CBF and SD_BOLD_, MSSD_BOLD_, SampEn_BOLD_ in the contralesional ROI.

The results of these correlation analyses are presented in Figure 8 and Table 3. The scatter plots in Figure 8A demonstrate a significant negative correlation of CBF with MSE at scales 7–20, and with relative delta power. This finding indicates that reduced blood flow in the perilesional cortex tends to be associated with higher entropy at longer time scales, and increased relative delta power. There was also a significant positive correlation between CBF and alpha and beta power, indicating that reduction in blood flow tends to be associated with attenuation of higher frequency oscillations in alpha and beta range (see Table 3). The correlations between CBF and measures of BOLD signal variability and complexity were not significant (see Figure 9A). There were no significant correlations present in the contralateral mirror ROI (see Figures 8B, 9B).

**Table 3:** **Pearson's correlations (2-tailed) between cerebral blood flow (CBF), multiscale entropy (MSE) at short (1–5) and long (7–20) time scales, and spectral band power, and measures of BOLD signal variability and complexity for stroke patients**.

	**CBF_LH**		**CBF_RH**	
	***r***	***p***	***r***	***p***
MSE_1–5	0.385	0.127	0.247	0.338
MSE_7–20	−0.485^*^	**0.049**	−0.381	0.132
Delta	−0.506^*^	**0.038**	−0.311	0.224
Theta	−0.135	0.606	−0.123	0.638
Alpha	0.524^*^	**0.031**	0.169	0.517
Beta	0.497^*^	**0.042**	0.331	0.195
SD_BOLD_	−0.099	0.706	0.023	0.929
MSSD_BOLD_	−0.088	0.738	0.020	0.940
SampEn_BOLD_	0.225	0.386	0.051	0.846

*Values extracted from the LH perilesional rim and the RH contralateral ROI*.

* *Correlation significant at the 0.05 level*.

*Significant correlations are shown in bold font*.

Overall these results suggest that MEG signal abnormalities, but not resting BOLD measures were associated with reduced perfusion values in the perilesional areas, and these measures were correlated across subjects.

### Relationship between MSE and spectral measures for stroke patients

In order to more fully understand the relationship between spectral band power and MSE at different time scales, we calculated Pearson's correlations between relative power at each frequency band and MSE measure. Table 4 shows correlations between MSE at short (1–5) and long (7–20) time scales vs. spectral power at different frequency bands for the stroke patients, using values averaged across voxels within the perilesional rim ROI for each patient. The analysis revealed that MSE at scales 1–5 was positively correlated with alpha and beta power, and negatively correlated with delta and theta power. In contrast, the relationship between MSE at scales 7–20 and spectral power went in the opposite direction. MSE at scales 7–20 correlated positively with delta and theta power, and negatively with alpha and beta power. These correlations support the findings described in Section Spectral Measures of MEG Resting Data: Effect of Aging and Stroke: perilesional areas exhibit decreased signal power and complexity at short time scales (higher frequencies), but increased power and complexity at longer time scales (lower frequencies). Spectral analyses and nonlinear complexity measures therefore reach similar conclusions based on the time scales involved.

**Table 4:** **Pearson's correlations (2-tailed) between multiscale entropy (MSE) at short (1–5) and long (7–20) time scales vs. spectral power at different frequency bands for stroke patients**.

	**MSE_1–5 LH**		**MSE_1–5 RH**	
	***r***	***p***	***r***	***p***
MSE_7–20	−0.804^**^	0.000034	−0.773^**^	0.000103
Delta	−0.849^**^	0.000004	−0.760^**^	0.000162
Theta	−0.713^**^	0.001	−0.897^**^	0.0000002
Alpha	0.679^**^	0.001	0.302	0.209
Beta	0.693^**^	0.001	0.692^**^	0.001
	**MSE_7–20 LH**		**MSE_7–20 RH**	
Delta	0.872^**^	0.000013	0.850^**^	0.000004
Theta	0.699^**^	0.001	0.808^**^	0.00003
Alpha	−0.625^**^	0.004	−0.372	0.117
Beta	−0.756^**^	0.00018	−0.723^**^	0.0005

*Values extracted from the LH perilesional rim and the RH contralateral ROI*.

** *Correlation significant at the 0.01 level*.

*Significant correlations are shown in bold font*.

## Discussion

Emerging trends in neuroimaging suggest that resting-state dynamics can identify localized abnormalities in cortex that is dysfunctional, but structurally intact, and that such findings can inform diagnoses and interventions. Although many modalities and analysis techniques have been successfully used to distinguish clinical populations from healthy controls, few studies have compared multiple methods to determine their relative sensitivity to different kinds of neural changes.

In the present study we examined the sensitivity of multiple measures of resting-state neural activity to the distinct changes associated with aging and stroke. For resting fMRI, we quantified signal variability (SD_BOLD_, MSSD_BOLD_) and sample entropy (SampEn_BOLD_). For resting MEG, we measured spectral changes in different frequency bands as well as MSE. In addition, we characterized the relationship between these measures and abnormalities in blood flow, which was quantified with pASL MRI.

The results suggest that reduced resting BOLD variability is a good predictor of aging. We found that BOLD variability was reliably reduced in older subjects, regardless of stroke, especially in the default mode regions, although variability increased in other regions. Similarly, older participants showed a widespread reduction in resting BOLD signal complexity (quantified using SampEn_BOLD_) and this decrease was not sensitive to the presence of stroke. In contrast, we found that MEG measures were more sensitive to the cortical abnormalities associated with stroke. MEG abnormalities manifested in perilesional tissue as increased slow-wave activity in the delta frequency bands, and reduced activity in higher bands including the alpha and beta bands. Stroke-related pathology was also associated with reduced MSE at shorter time scales (MSE scales 1–5), and with increased MSE at the longer scales (7–20). In addition, we observed a general effect of aging which manifested in the speeding of electrophysiological activity, and it was in the opposite direction to the slowing observed in stroke patients (decreased MSE at scales 7–20, decreased delta power, increased alpha and beta power). Furthermore, MEG signal abnormalities, but not resting BOLD measures, were associated with reduced perfusion values in perilesional areas, and these measures were correlated across stroke patients.

### fMRI measures of spontaneous neural activity

Our finding that aging is associated with decreased variability and complexity in the BOLD signal is consistent with previous findings in the literature (Garrett et al., 2010, 2011, 2013a). It has been proposed that increases in signal variability may represent a more complex neural system that is able to more efficiently process and respond to a wider range of stimuli. This greater “dynamic range” allows the system to better adapt responses to unexpected external events, and to more easily transition between different states. Another link between BOLD signal variability and cognitive performance was reported by Protzner et al. (2013), who observed that increased SD_BOLD_ was correlated with verbal memory retention in patients with temporal lobe epilepsy.

It has been suggested that some forms of brain variability derive from coherent spontaneous fluctuations throughout the cortex, with greater variability reflecting greater coherence between different brain regions (Fox et al., 2006; Nir et al., 2008). Other accounts propose that brain variability may be a function of connectivity between different brain areas. Older adults often exhibit reductions or alterations in both structural and functional brain connectivity (Grady et al., 2010). Thus, it is possible that the reduced BOLD variability found in our older adults may reflect decreased network complexity and connectivity in the default mode regions with increasing age. Another a related possibility is that reduced BOLD variability in older adults reflects age-related losses in white matter integrity or synaptic density.

Interestingly, we also found increased BOLD signal variability in superior frontal, inferior temporal, and cerebellar regions in older adults. Similar age-related bidirectional effects were previously reported by Garrett et al. (2010, 2011). Although it is not clear what the increases reflect, it has been suggested that they relate to a compensatory process. In the context of the present results it may reflect a process that attempts to counteract the age-related reduction of resting brain network complexity and integration. In some comparisons between stroke patients and controls we found that SD_BOLD_ values tended to be high inside the lesions themselves. This increase was observed because the raw fMRI signal strength in the lesions was much higher than the rest of the brain. Normalizing the signal to a whole-brain mean did not reduce this effect. We also considered normalizing each voxel to it's own percent signal change, but this resulted in strong artifacts in SD measures, because noise at the edge of the brain became strongly amplified. This is one limitation of the SD measure when applied to stroke brains. In studies of individuals, high signal within the lesions must be disregarded.

In addition to reduced signal variability in the default mode regions, we also found that, compared to the younger group, older adults showed a widespread reduction in the SampEn_BOLD_ of the resting fMRI signal. These results are broadly consistent with the idea that increased BOLD variability reflects a more complex and sophisticated neural system, and suggest that reduction in resting brain signal complexity is associated with the normal aging process. Entropy of brain signals in relation to aging has been examined in previous studies. Yang et al. (2013) used MSE analysis to quantify the complexity of BOLD activity at rest. They found that the complexity of spontaneous BOLD signal was reduced in older adults, and that this decrease in complexity in the default mode network areas was related to lower cognitive performance. In a more recent study, Sokunbi (2014) applied SampEn analysis to resting-state data obtained from young and elderly adults and found that whole brain SampEn values decreased with age.

Convergent with previous findings, the present results indicate that, to the extent that BOLD signal variability and entropy measures index neural complexity, integration, and connectivity between brain regions, they could serve as markers of neural processing efficiency. The results suggest that estimates of complexity and variability of spontaneous BOLD signal can reveal subtle changes associated with the aging process. Surprisingly, BOLD variability and complexity measures were not sensitive to the neural abnormalities occurring in perilesional cortex in chronic stroke patients. The reason for this lack of sensitivity is not clear. The neural changes occurring in perilesional tissue are different than those associated with normal aging, and therefore might require different measures to detect them. One possibility is that pathological changes following stroke might result in increased BOLD variability in some cases, and decreased variability in others, resulting in no consistent significant effect on the group level.

### MEG measures of spontaneous neural activity

In contrast to BOLD signal measures, we found that MEG measures were more sensitive to the functional abnormalities associated with stroke. In comparisons to both control groups, stroke patients exhibited clear alterations of electrophysiological activity in the areas surrounding the lesion. These changes took the form of a “slowing” of the signal, shifting more toward slower frequencies in the delta band, and away from the beta band. Additionally, stroke patients showed changes in MSE that were scale dependent. Perilesional tissue exhibited reduced MSE at time scales 1–5, but increased MSE at the longer scales (7–20).

The MEG results of the present study extend our previous findings (Chu et al., 2015) showing that aging is associated with changes in spectral power and MSE (but only at longer time scales). In particular, older adults showed a widespread bilateral decrease in delta power, and an increase in alpha and beta oscillatory activity. Aging was also associated with decreased MSE values at the longer time scales (7–20, corresponding to periods of 33–96 ms). In contrast, MSE at the shorter scales (1–5, corresponding to 4.8–24 ms) was specifically sensitive to stroke and not aging.

Thus, it appears that in some cortical regions older adults show a general speeding of electrophysiological activity, which is in the opposite direction of the slowing associated with perilesional tissue in stroke. This increase in the alpha and beta oscillatory activity in the superior frontal and anterior frontal regions appears to be a general index of the aging process rather than a sign of cortical pathology. The present finding supports previous reports of age-related speeding of electrophysiological activity. Holschneider and Leuchter (1995) reported resting-state increases in EEG beta power with age, whereas it was decreased in patients with dementia. Similarly, Bruce et al. (2009) found decreased relative delta and increased relative beta associated with aging. Similarly, Vlahou et al. (2014) observed reduced delta and theta in older subjects using MEG. Our finding of age related decreases in MSE at longer scales is similar to findings reported by McIntosh et al. (2014). In that study, aging was associated with increased entropy at short scales and decreased entropy at longer scales.

It has been suggested that age-related speeding of the frequency spectrum may reflect compensatory activity in response to decreased nerve conduction velocities (Hong and Rebec, 2012). In the present study, the findings of age-related alpha and beta power increase, together with increased BOLD signal variability in the frontal regions, are consistent with this hypothesis.

Reduced MSE values have been considered a general indicator of dysfunctional processing (Park et al., 2007; Poza et al., 2007; Beharelle et al., 2012). In the present study, findings of altered entropy values in the regions surrounding the lesion suggest that MSE may be a sensitive indicator of perilesional cortical dysfunction. This perilesional tissue appears to be structurally intact, but—as indicated by the reduced entropy—it is processing information in a suboptimal way. We note, however, that MSE is not invariably reduced at all time scales in dysfunctional perilesional tissue. Rather, it is decreased at finer time scales and increased at coarser ones. Thus, studies using MSE as a diagnostic/prognostic indicator should be careful to take time scale into account. Our results indicate that linear spectral analyses and MSE (a nonlinear measure) have similar sensitivity to the altered physiology in perilesional tissue, and are highly correlated with each other. Cortical tissue affected but not destroyed by stroke is characterized by a shift to more activity at longer time scales (lower frequencies) and less at shorter time scales (higher frequencies). In our previous study (Chu et al., 2015), we found that MSE at fine time-scales was correlated with the ratio of high-frequency to low-frequency power, but that study did not examine MSE at longer time scales because it was based on shorter segments of data, taken from the inter-trial interval of a cognitive task. In the present study, using true resting data, we observed that the correlation with spectral power reverses itself at longer time scales.

Overall, the present results indicate that both spectral slowing and altered MSE values can serve as sensitive indicators of neuronal damage associated with stroke. Furthermore, the combination of these measures can reveal the extent of perilesional tissue that is structurally preserved but functionally compromised. The present findings are consistent with those reported in the MEG and EEG literature. Other MEG studies have found pathological shifts toward low frequency activity in the affected hemisphere, in both acute and chronic stroke patients (Butz et al., 2004; Meinzer et al., 2004; Laaksonen et al., 2013; Chu et al., 2015). Convergent with the present findings, Tecchio et al. (2005, 2006)—using analysis of MEG sensor data—reported increases in delta and theta band power and decreases in beta and gamma power on the side of the lesion, as well as a decrease in spectral entropy when compared to controls and the unaffected hemisphere.

### Relationship to blood flow

Our results suggest that perilesional slowing and reduced entropy (MSE scales 1–5) of spontaneous electrical activity are reliable indicators of neuronal pathology and dysfunctional information processing. However, underlying causes of this abnormal activity are not well understood. One potential mechanism could be related to chronic hypoperfusion, which can compromise the functional integrity of the cortex without causing neuronal necrosis. It is possible that ischemic damage at the time of the stroke resulted in some neural cell death and synaptic loss, but left enough living cells to maintain the basic structural integrity of the tissue. This mechanism is also consistent with our finding that reduced blood flow is correlated with both reduced MSE (scales 1–5) and increased delta power. Tissue that is less metabolically active will receive less blood flow due to mechanisms of neurovascular coupling. Chronic dysfunction in structurally intact cortex has been demonstrated by nuclear imaging studies in chronic stroke, which have revealed associations between hypoperfusion, hypometabolism, and markers of neuronal injury, such as reduced benzodiazepine receptor density (Sasaki et al., 1997; Oku et al., 2010).

Consistent with our findings, several EEG studies in acute and subacute stroke found a relationship between reduced CBF and increased delta power (Claassen et al., 2004; Jordan, 2004; Friedman and Claassen, 2010; Finnigan and van Putten, 2013). However, the effects of altered CBF on oscillatory activity and its relationship to chronic stroke have not been well investigated. In the present study, we characterized the relationships between spontaneous electrical activity and chronic hypoperfusion. Our results show that the brain regions that exhibited shifts to greater power and complexity at longer time scales (lower frequencies) also showed reduced perfusion values. This relationship between abnormalities in brain function and blood flow data was strongest in the perilesional left hemisphere regions.

In addition, our results provide evidence of age-related changes in CBF. In comparison to young controls, older adults exhibited reduced blood flow along the occipital and superior parietal and frontal regions bilaterally, whereas stroke damage was associated with reduced CBF values along the temporal gyrus and precentral areas surrounding the infarct. These blood flow changes in older adults seem to be specific to the aging process, and distinct from the cortical damage associated with the stroke lesion. Our findings are in agreement with previously reported patterns of age-related decreases in CBF (Chen et al., 2011, 2013).

One remaining question from this work concerns the causality of the relationship between abnormal electrical activity and hypoperfusion in perilesional tissue. The relationship between these measures suggests that perilesional dysfunction may be reversible. Investigation of interventions that may normalize perilesional activity would help to clarify the relationship, and would have obvious clinical applications for the treatment of cognitive and motor deficits in stroke. For example, the GABA-receptor positive allosteric modulator drug, zolpidem, has been shown to induce increased blood flow in minimally conscious patients (Brefel-Courbon et al., 2007; Nyakale et al., 2010), and in one case report, an aphasic stroke patient (Cohen et al., 2004). These blood flow increases have been accompanied by behavioral improvement, and the therapeutic response to this drug has also been associated with a normalization of abnormal slow-wave activity (Hall et al., 2010; Williams et al., 2013). Noninvasive brain stimulation techniques such as TMS and TDCS have also been shown to produce functional gains in post-stroke aphasia (Martin et al., 2009; Baker et al., 2010; Hamilton et al., 2011; Holland and Crinion, 2012), but the physiological changes corresponding to these gains are as yet poorly understood. Our findings suggest that reduced MEG signal complexity and reduced blood flow may index the physiological deficits in chronic stroke, and that these imaging modalities should be further explored to assess the success of potential interventions.

One limitation of the study is that our sample of stroke patients was heterogeneous with respect to the aphasia type and lesion size, and it was not possible within our sample size to define subsamples based on more specific phenotypes. However, all of the patients had their lesion in the left hemisphere, involving perisylvian language regions. Thus, general differences between the affected left hemisphere and the undamaged right hemisphere are valid despite the variability. Furthermore, we supplemented the voxel-wise analysis with individualized perilesional ROIs in each subject, compared with healthy tissue occupying the same areas in the contralesional hemisphere (Figures 5, 8, 9). These results do lead to a consistent pattern of abnormalities in perilesional cortex. The voxel-wise whole-brain maps confirm that the abnormalities are most prominent in the border zone of the lesions, rather than being distributed throughout the damaged left hemisphere. Nonetheless, statistical power in a study like this is reduced by the large heterogeneity in lesion location across the participants. Some whole-brain maps had large FDR *q*-values, indicating poor localization value of the maps. The general pattern of results for all of those maps was still present at a higher voxel-wise threshold of *p* < 0.001 uncorrected (Supplementary Information). However, ultimately the limitations of group analysis for perilesional tissue abnormalities are somewhat irrelevant for their ultimate clinical application. We believe that the techniques presented in this study will be most useful at the individual subject level. In our previous MEG study in aphasic stroke patients, we demonstrated that maps can be made for single subjects highlighting perilesional abnormalities (see Figure 5 of Chu et al., 2015). In future work, we intend to target such dysfunctional cortex for intervention with noninvasive brain stimulation. For such treatments, mapping and quantification of abnormal physiological activity on the individual level will be essential.

## Conclusions

In the present study, we characterized spontaneous neural activity using MEG and fMRI. We aimed to assess the utility and sensitivity of signal variability and complexity measures as indicators of age-related changes and perilesional dysfunction in chronic stroke. Our results demonstrate that BOLD measures of variability and complexity are associated with aging-related changes, whereas measures of spontaneous MEG activity are better indicators of neuronal dysfunction associated with stroke. Furthermore, shifts of the MEG signal toward dynamics on longer time scales (both in oscillatory power and entropy) were associated with reduced blood flow in the brain regions adjacent to the lesion. We suggest that these measures may be useful indicators of cortical dysfunction that is potentially reversible with treatment, and may be used to assess the effectiveness of interventions.

## Author contributions

Conceived and designed the study: AK, JM. Acquired imaging and cognitive data: AK, TD. Performed neuropsychological assessments: RJ, TD, AK. Analyzed the data: AK, JM, RC. Wrote pASL MRI acquisition sequences and ASL analysis scripts: JC, YK. Wrote the paper: AK, JM, TD.

### Conflict of interest statement

The authors declare that the research was conducted in the absence of any commercial or financial relationships that could be construed as a potential conflict of interest. The reviewer IC and handling Editor declared their shared affiliation, and the handling Editor states that the process nevertheless met the standards of a fair and objective review.
