# Supplementary material for: Identifying Dysfunctional Cortex: Dissociable Effects of Stroke and Aging on Resting State Dynamics in MEG and fMRI
Source: Front Aging Neurosci. 2016 Mar 3;8:40. doi: 10.3389/fnagi.2016.00040 (PMC4776400; doi:10.3389/fnagi.2016.00040)
Supplement: Supplementary file 1 [file Image1.pdf]

Supplementary Materials

In addition to the standard deviation (SD_{BOLD}; Garrett et al., 2011) of the BOLD signal, we also computed mean squared successive difference (MSSD; Leo et al., 2012; Mohr, & Nagel, 2010), as a measure of signal variability. The group comparison maps of $MSSD_{BOLD}$ values are shown in Figure S1. Locations of significant clusters are listed in Table S2.

BOLD signal variability ($MSSD_{BOLD}$)*: Effect of aging and stroke*
The comparison of $MSSD_{BOLD}$ values for stroke patients with young controls revealed decreased variability for patients in the precuneus and cuneus, bilaterally. The analysis also revealed areas of increased variability for the stroke patients along the left and right superior frontal gyrus and medial frontal areas, as well as anterior cingulate gyrus, fusiform gyrus and cerebellum, bilaterally. However, the comparison between age-matched controls and stroke patients did not detect significant differences in variability.

The comparison of $MSSD_{BOLD}$ maps for age-matched controls with young controls revealed decreased BOLD signal variability for older participants in the posterior cingulate gyrus, cuneus and precuneus, bilaterally. The analysis also revealed areas of increased variability for the older adults in the left and right superior frontal gyrus, as well as cingulate gyrus, and cerebellum, bilaterally.

This pattern of results is very similar to what was observed for SD_{BOLD} values. The results revealed significant reductions of resting state BOLD variability in medial parietal-occiptal regions, and increases elsewhere, in older participants compared to younger participants, regardless of stroke status. Stroke patients did not show significant changes compared to age-matched controls.

Figure S1. **Group comparison maps of $MSSD_{BOLD}$ values.** (STP = stroke, YC = young controls, AM = age-matched). For comparisons with stroke patients the results were overlaid on top of an artificially darkened anatomical image representing the lesion distribution across patients. Darker colours represent greater lesion overlap in these areas. The statistical maps were thresholded at voxelwise threshold of $p < .01$, with a minimum cluster size of 20 voxels. False discovery rates (q-value) are indicated for each map. Blue colors reflect the decrease in BOLD MSSD, and red colors reflect increases present in significant voxels within each activation map. **(A)** Stroke vs. young controls. **(B)** Stroke vs. age-matched controls. **(C)** Age-matched controls vs. young controls.

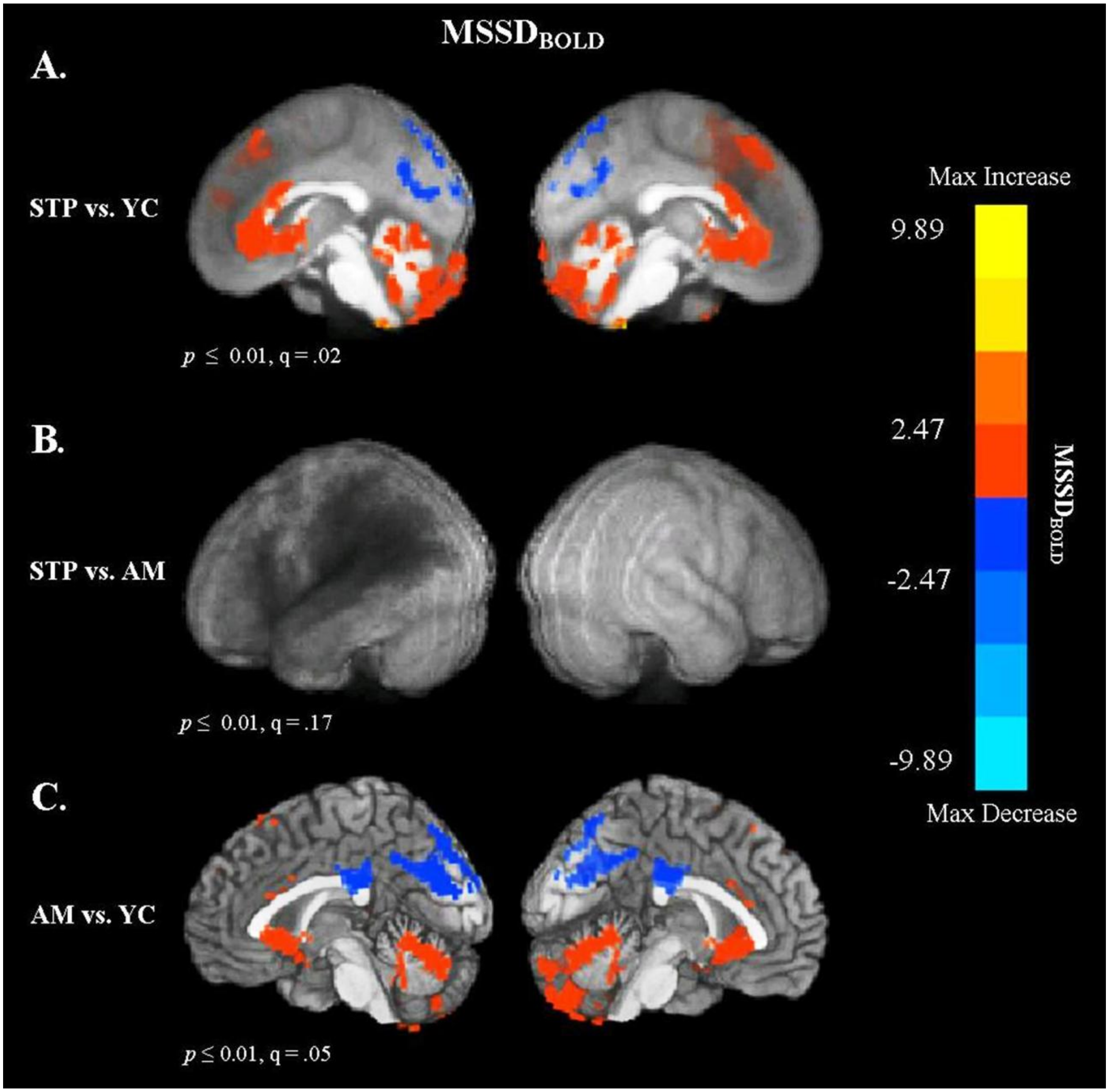

Higher-threshold maps.

Some of the whole-brain voxel-wise maps shown in the main text had patterns of activity significant at a voxel-wise threshold of $p < .01$, but with a large FDR of $q > .05$. To confirm the reliability of activation in maps showing such patterns, we conducted a supplementary analysis at a higher voxel-wise threshold of $p < .001$ uncorrected. In all cases, the pattern of results was unchanged – we still saw significant effects at .001 where we saw them at .01, and of course comparisons without significant activation remained insignificant at the higher threshold. The supplementary maps are shown here.

Figure S2. **Between group voxel-wise contrast maps of MSE** (STP = stroke, YC = young controls, AM = age-matched). The statistical maps were thresholded at a voxelwise threshold of p = .001. False discovery rates (q-value) are indicated for each map. These are the same maps shown in Figure 3, but at a higher voxel-wise threshold. **(A)** The between group t-test comparison maps of MSE scales 1-5. **(B)** The between group t-test comparison maps of MSE scales 7-20.

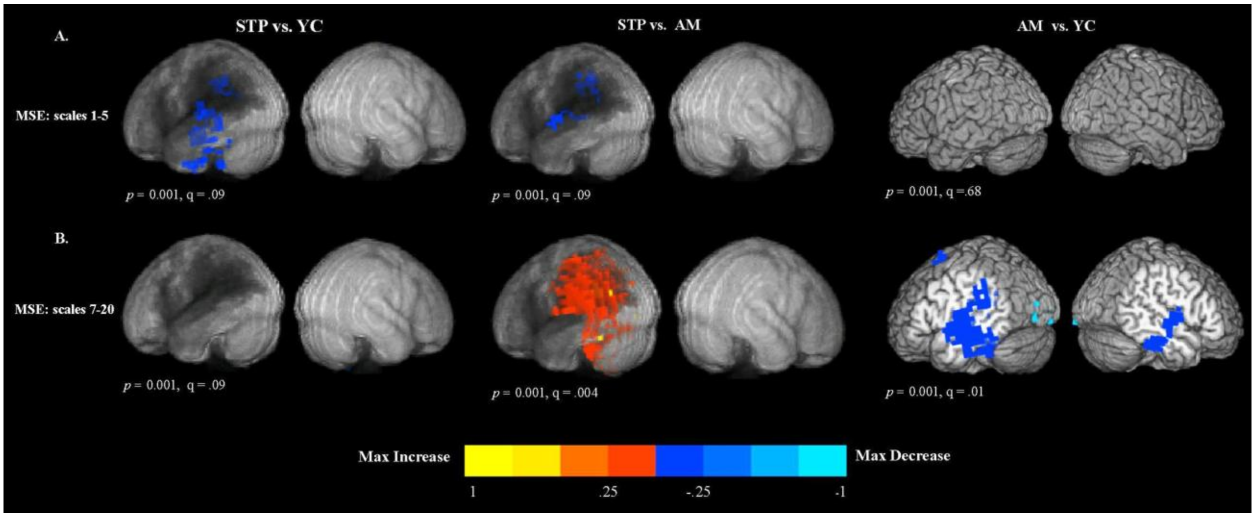

Figure S3. **Group comparison CBF maps obtained with pulsed Arterial Spin Labelling MRI.** (STP = stroke, YC = young controls, AM = age-matched). The statistical maps were thresholded at a voxelwise threshold of p = .001. Blue colors reflect a decrease in CBF. These are the same maps shown in Figure 6, but at a higher voxel-wise threshold. **(A)** CBF for Stroke patients vs. Young controls. **(B)** Stroke patients vs. Older Age matched controls. **(C)** Older controls vs. Young Controls.

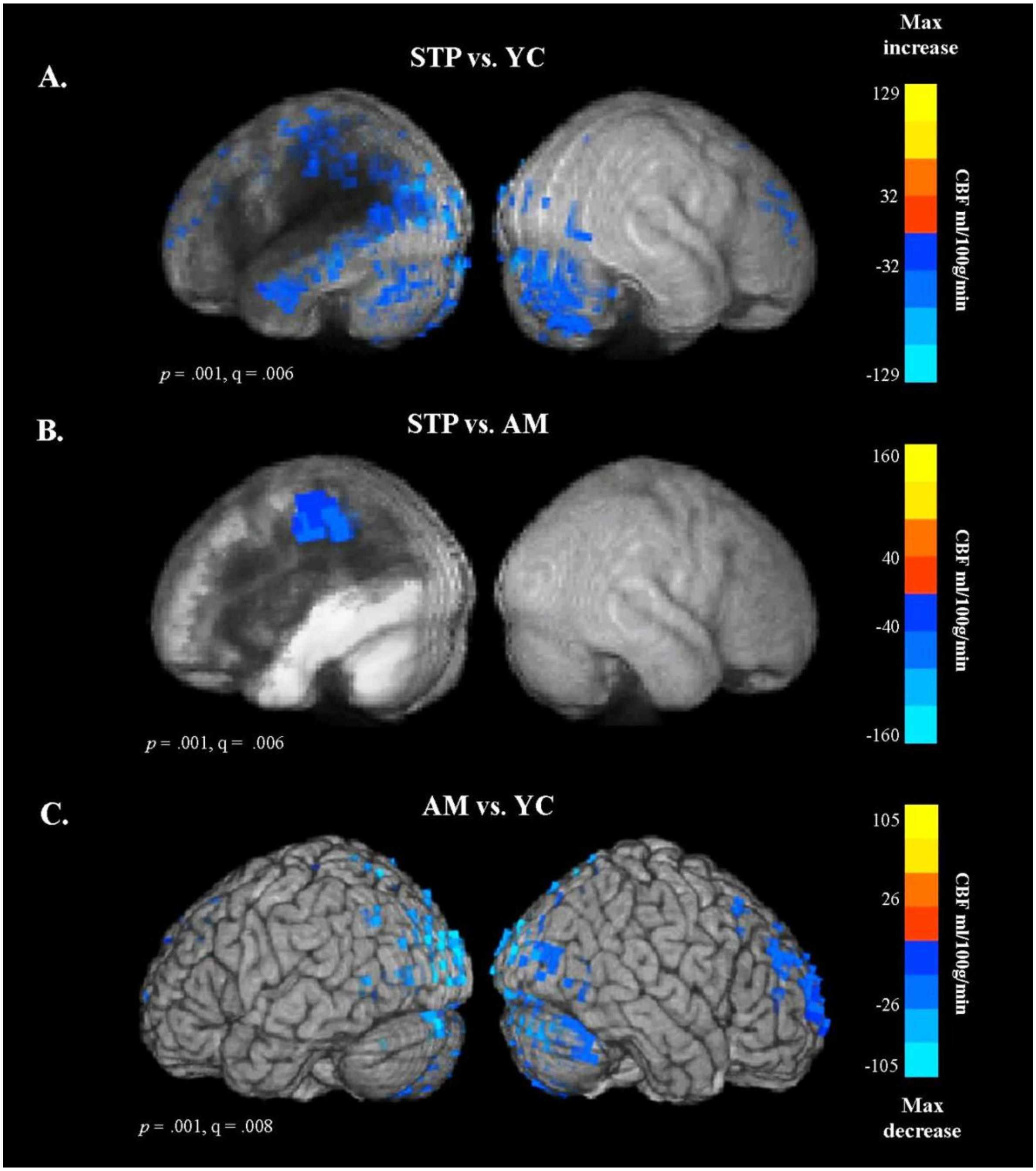

Figure S4. **Relationship between cerebral blood flow (CBF) and the MSE and spectral measures obtained from resting MEG data.** The maps show whole-brain, voxel-wise Spearman's rank-order correlations between CBF and MEG measures (MSE scales 1-5, MSE scales 7-20, and relative spectral band power). The statistical maps were thresholded at a voxelwise threshold of p = .001. These are the same maps shown in Figure 7, but at a higher voxel-wise threshold. **(A)** Spearman's rank-order correlation map between CBF and MSE scales 1-5. **(B)** Spearman's rank-order correlation map between CBF and MSE scales 7-20. **(C)** Spearman's rank-order correlation map between CBF and relative delta power. **(D)** Spearman's rank-order correlation map between CBF and relative alpha power. **(E)** Spearman's rank-order correlation map between CBF and relative beta power.

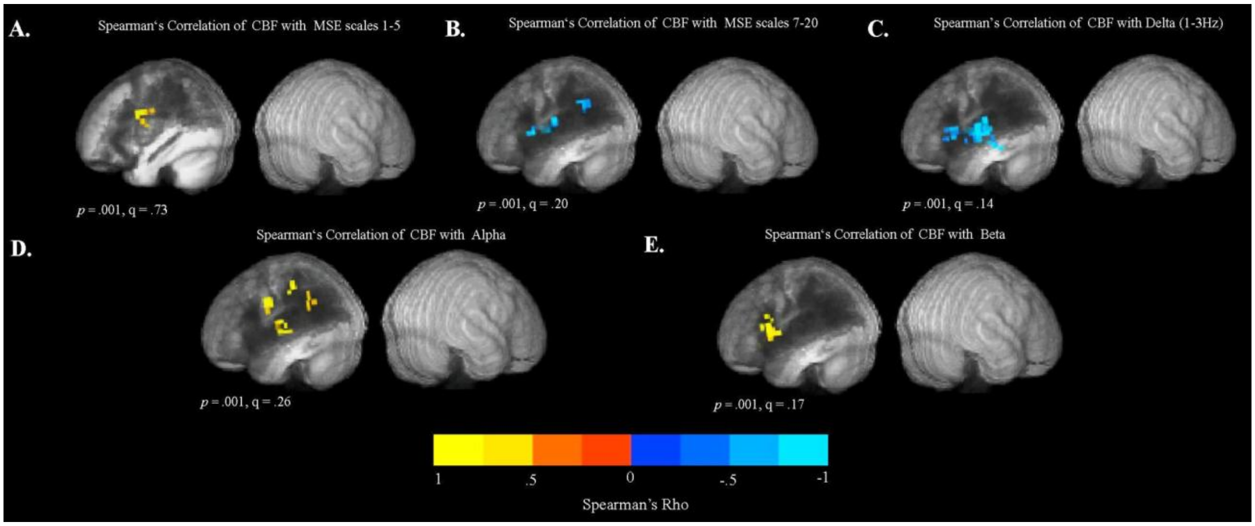

Appendix

This section contains the tables listing significantly activated regions. We did not to list activated regions for BOLD Sample entropy (displayed in Figure 2B) and MEG resting measures (MSE, spectral results shown in Figures 3 and 4) because these effects had widespread distribution in the left hemisphere.

Table S1. **BOLD Signal Variability (SD_{BOLD}) between group comparisons**. Locations of significant clusters and cluster extent, MNI coordinates, and cluster sizes.

	Region Label	Hemisphere	MNI coordinates			Vol_Voxels
**STP vs YC**			x	y	z	
**SD_{BOLD}** decreases						
	cuneus, BA17/18	R	2	-82	8	453
	precuneus	R	10	-64	30	
	cuneus	L	-4	-80	8	
	precuenus	L	-4	-68	35	
	posterior cingulate	R/L	0	-28	26	201
	posterior cingulate	R	2	-28	26	
	posterior cingulate	L	-4	-28	26	
	IPL, BA 39/40	L	-54	-60	26	67
	precuneus, BA7/19	R	2	-84	44	56
	precuneus	L	-2	-78	50	
	precuneus, BA 7/SPL	L	-24	-70	44	32
	medial frontal	R	0	54	-4	45
	medial frontal	R	2	56	-4	
	medial frontal	L	-2	52	-4	
**SD_{BOLD}** increases	cerebellum	L	-10	-34	-58	9184
	cerebellum	L	-22	-48	-58	
	cerebellum	R	12	-42	-58	
	cerebellum	R	44	-46	-44	
	fusiform gyrus	L	-50	-40	-30	
	fusiform gyrus	R	42	-38	-26	
	anterior cingulate gyrus	L	-6	24	-4	
	anterior cingulate gyrus	R	2	22	-4	
	superior frontal	R	14	14	54	
	superior frontal	L	-6	18	60	
	IFG, BA47	R	26	8	-16	976
	IFG	R	32	12	-16	
	cingulate gyrus	R	18	-28	30	131
	right medial frontal	R	18	-24	52	
	IFG, BA47/45	L	-24	32	-6	26
**STP vs AM**						

SD_{BOLD} decreases		no significant clusters				
SD_{BOLD} increases	IPL	L	-46	-30	26	78
	postcentral gyrus	L	-40	-22	28	
**AM vs YC**						
SD_{BOLD} decreases	precuneus	R	3	-84	45	883
	precuneus	R	4	-70	28	
	precuneus	L	-2	-82	45	
	precuneus	L	-2	-73	28	
	posterior cingulate	R	4	-31	27	
	posterior cingulate	L	-3	-31	27	
	IPL, BA39	R	51	-63	45	114
	IPL, BA40	L	-60	-36	27	40
	cuneus	L	-24	-87	24	39
	precuneus	L	-42	-72	36	27
	middle occipital/BA19/cuneus	R	36	-84	15	48
	IFG/BA47	L	-54	18	0	45
	middle frontal	L	-24	-3	57	23
	superior/middle frontal	L	-27	27	57	22
	middle frontal/IFG	L	-48	45	-3	53
	MTG/STG/BA22	L	-57	-42	3	65
	posterior cingulate	L	-12	-60	3	20
SD_{BOLD} increases	cerebellum	R	9	-60	-66	1892
	cerebellum	L	-11	-59	-63	
	anterior cingulate	R/L	0	9	-9	152
	anterior cingulate	L	-4	13	-9	
	anterior cingulate	R	2	11	-4	
	middle cingulate	R	12	9	30	82
	anterior cingulate	L	-6	8	29	80
	middle cingulate/ postcentral gyrus	R	24	-30	45	79
	parahippocampal	L	-27	6	-18	62
	medial fontal/superior frontal	R/L	0	66	-12	33
	middle cingulate	L	-21	-27	39	33
	fusiform gyrus	L	-45	-39	-30	28

IPL: Inferior parietal lobule, SPL: superior parietal lobule, IFG: inferior frontal gyrus, MTG: middle temporal gyrus, STG: superior temporal gyrus.
Vol_Voxels: volume given in voxels. Voxel size 3x3x3 mm.

Table S2. **BOLD Signal Variability ($MSSD_{BOLD}$) between group comparisons**. Locations of significant clusters and cluster extent, MNI coordinates, and cluster sizes.

	Region label	Hemisphere	MNI coordinates			Vol_Voxels
			x	y	z	
**STP vs YC** **$MSSD_{BOLD}$** decreases						
	cuneus	R	2	-76	8	76
	cuneus	L	-2	-82	8	
	precuneus	L	-2	-72	28	
	cuneus	R	6	-102	0	29
	precuneus	R	4	-68	28	
**$MSSD_{BOLD}$** increases						
	cerebellum	L	-34	-50	-46	15542
	cerebellum	R	24	-50	-46	
	fusiform gyrus	L	-42	-38	-20	
	fusiform gyrus	R	50	-52	-11	
	cerebellum	R/L	0	-46	-17	22
	inferior parietal	R	38	-30	24	36
	superior/middle frontal	L	-40	38	38	33
	medial frontal	L	-20	30	34	
	medial frontal	R	16	30	34	
	superior frontal	R	16	42	38	
	anterior cingulate	R	16	24	-8	
	anterior cingulate	L	-16	26	-8	
**STP vs AM** **$MSSD_{BOLD}$** decreases		no significant clusters				
**$MSSD_{BOLD}$** increases		no significant clusters				
**AM vs YC** **$MSSD_{BOLD}$** decreases	precuneus	R	3	-84	42	180
	precuneus	R/L	0	-81	39	
	precuneus	L	-1	-75	24	
	cuneus	L	-1	-84	15	
	cuneus	R	6	-83	19	
	posterior cingulate	R/L	0	-2	27	53
	posterior cingulate	R	4	-33	27	
	posterior cingulate	L	-2	-34	27	

**$MSSD_{BOLD}$** increases	cerebellum	L	-18	-46	-51	2096
	cerebellum	R	17	-48	-63	
	superior frontal	R	9	21	66	703
	superior frontal	L	-9	11	58	
	medial frontal	R	13	-3	58	
	anterior cingulate	R	13	10	28	
	cingulate gyrus	L	-3	6	30	334
	cingulate gyrus	R	8	8	30	
	IFG/BA45	R	39	33	3	75
	parahippocampal gyrus	L	-24	6	-18	257
	anterior cingulate	R	11	12	-9	
	anterior cingulate	L	-9	17	-9	

IFG: inferior frontal gyrus

Table S3. **Cerebral Blood Flow (CBF) between group comparisons**. Locations of significant clusters and cluster extent, MNI coordinates, and cluster sizes.

	Region Label	Hemisphere	MNI coordinates			Vol_Voxels
**STP vs YC**			x	y	z	
	ITG	L	-62	-50	-14	2831
	MTG	L	-60	-32	-12	
	fusiform	L	-48	-60	-20	
	precuneus	L	-16	-66	48	
	cuneus	L	-16	-96	8	
	IPL/SMG	L	-36	-50	38	
	SPL	L	-24	-72	44	
	middle occipital gyrus	L	-26	-92	16	
	postcentral gyrus	L	-36	-38	56	
	precentral gyrus	L	-38	-14	52	
	middle frontal gyrus, BA8	L	-24	10	54	
	superior frontal	L	-24	20	54	
	cuneus	R	24	-92	6	
	middle occipital gyrus	R	42	-84	6	
	middle frontal gyrus, BA8/9	R	42	30	40	266
**STP vs AM**						
	IPL/BA40	L	-32	-50	40	206
	SPL	L	-32	-56	48	
	precentral gyrus	L	-32	-16	46	
	MTG	L	-62	-40	-14	35
	MTG/BA39	L	-48	-70	10	31
	fusiform gyrus	L	-62	-10	-30	22
**AM vs YC**						
	cuneus	R	7	-91	7	1867
	cuneus	L	-13	-96	7	
	middle occipital	L	-34	-90	7	
	inferior occipital	L	-34	-75	-11	
	cerebellum	L	-34	-62	-35	
	precentral	L	-29	-26	68	
	postentral	L	-16	-54	69	
	cerebellum	R	16	-77	-27	
	precuneus	R	16	-75	46	

postcentral	R	16	-55	69	
SPL	L	-16	-79	57	
SPL	R	22	-71	52	
middle frontal	L	-38	24	50	679
medial frontal	R/L	0	31	47	
superior frontal	R	15	44	47	
superior frontal	L	-9	47	47	
middle occipital	R	57	-66	-10	46
inferior temporal	L	-58	-31	-25	27

IPL: Inferior parietal lobule, SPL: superior parietal lobule, IFG: inferior frontal gyrus, MTG: middle temporal gyrus, STG: superior temporal gyrus, ITG: inferior temporal gyrus, SMG: supramarginal gyrus.

Table S4. Results of Spearman's rank-order correlations between MEG measures and CBF. Locations of significant clusters and cluster extent, MNI coordinates, and cluster sizes.

Region Label	Hemisphere	MNI coordinates			Vol_Voxels
**MSE (scales 1-5) vs CBF**		x	y	z	
precentral	L	-38	-6	26	45
IFG	L	-44	-4	26	
anterior cingulate	L	-22	20	26	28
**MSE (scales 7-20) vs CBF**					
insula	L	-42	-20	16	198
IFG	L	-52	8	16	
middle frontal	L	18	-16	66	57
middle temporal/AG	L	-38	-66	20	44
postcentral	L	-32	-26	50	21
**Delta power vs CBF**					
left insula	L	-38	-16	10	45
STG	L	-62	-16	2	
**Alpha power vs CBF**					
postcentral gyrus	L	-62	-30	20	117
precentral	L	-62	-12	32	
STG	L	-62	-28	6	
superior frontal	L	-18	34	40	38
parietal lobule/BA 40	L	-52	-36	46	26
**Beta power vs CBF**					
insula	L	-32	20	6	97
IFG (BA45)	L	-44	20	6	
IFG (BA44/45)	L	-52	10	20	44

IFG: inferior frontal gyrus, AG: angular gyrus, STG: superior temporal gyrus
Vol_Voxels: volume given in voxels. Voxel size 5x5x5 mm.
